# A Systematic Literature Review on Machine and Deep Learning Approaches for Detecting Attacks in RPL-Based 6LoWPAN of Internet of Things

**DOI:** 10.3390/s22093400

**Published:** 2022-04-29

**Authors:** Taief Alaa Al-Amiedy, Mohammed Anbar, Bahari Belaton, Arkan Hammoodi Hasan Kabla, Iznan H. Hasbullah, Ziyad R. Alashhab

**Affiliations:** 1 National Advanced IPv6 Centre (NAv6), Universiti Sains Malaysia, Gelugor 11800, Penang, Malaysia; taiefalamiedy@student.usm.my (T.A.A.-A.); arkan@student.usm.my (A.H.H.K.); iznan@nav6.usm.my (I.H.H.); zalashhab@student.usm.my (Z.R.A.); 2 School of Computer Sciences, Universiti Sains Malaysia, Gelugor 11800, Penang, Malaysia; bahari@usm.my

**Keywords:** 6LoWPAN, Internet of Thing (IoT), IPv6, Low Power and Lossy Network (LLN), Machine Learning (ML), Deep Learning (DL), RPL security and threats, Systematic Literature Review (SLR)

## Abstract

The IETF Routing Over Low power and Lossy network (ROLL) working group defined IPv6 Routing Protocol for Low Power and Lossy Network (RPL) to facilitate efficient routing in IPv6 over Low-Power Wireless Personal Area Networks (6LoWPAN). Limited resources of 6LoWPAN nodes make it challenging to secure the environment, leaving it vulnerable to threats and security attacks. Machine Learning (ML) and Deep Learning (DL) approaches have shown promise as effective and efficient mechanisms for detecting anomalous behaviors in RPL-based 6LoWPAN. Therefore, this paper systematically reviews and critically analyzes the research landscape on ML, DL, and combined ML-DL approaches applied to detect attacks in RPL networks. In addition, this study examined existing datasets designed explicitly for the RPL network. This work collects relevant studies from five major databases: Google Scholar, Springer Link, Scopus, Science Direct, and IEEE Xplore^®^ digital library. Furthermore, 15,543 studies, retrieved from January 2016 to mid-2021, were refined according to the assigned inclusion criteria and designed research questions resulting in 49 studies. Finally, a conclusive discussion highlights the issues and challenges in the existing studies and proposes several future research directions.

## 1. Introduction

Internet of Things (IoT) has become one of the most important elements of the Information and Communication Technology (ICT) revolution. IoT plays a significant role in connecting smart objects anytime, anywhere, and any service through any network. The entire world looks forward to creating a new smart world that changes people’s lifestyles and how things work in our world [1,2]. Consequently, the IoT affects everything from our lifestyle to how we live in this era of technological convergence and inter-connectivity. The IoT incorporates the linkage of human culture—our “things”—considering the interconnection of our computerized data framework—”the Internet”. The paradigm of IoT has spread all over the world. It foresees the networking of billions to trillions of smart things around us, specifically ordinary things that are extraordinarily traceable, addressable, and have the ability to collect, store, analyze, and communicate data about themselves and their physical surroundings [3,4].

In addition, IoT objects or devices may connect bidirectionally for data exchange over the Internet. Furthermore, it is highly advantageous to people since it optimizes their time and boosts productivity. Consequently, we can reinvent ourselves while simultaneously making the world and our lives smarter with the help of IoT. Moreover, the benefits of IoT are nearly limitless, and its applications are transforming how we work and live by exchanging time and assets and opening up new prospects for growth and development [5]. Moreover, a recent forecast from International Data Corporation (IDC), a well-known provider of industry intelligence, predicts that by 2025, there will be around 41.6 billion connected IoT devices/things (a combination of sensors, machines, cameras, etc.), generating around 79.4 zettabytes of data. The forecast was based on an analysis spanning the years from 2018 to 2025, and during that period, they expected IoT devices to grow at a Compound Annual Growth Rate (CAGR) of 28.7%.

The various IoT sensors deployed in different environments are responsible for collecting data in the network and sending them to the backbone servers and control centers for further analysis to assist in decision making. The number of IoT-based applications has increased exponentially in recent years, and while many are still in the early research stage, many economically attractive application scenarios already exist that span several domains [6]. Some of these applications include smart firefighting for forest fire detection and personal protective equipment monitoring; smart manufacturing for monitoring air quality, temperature, and cyber-physical systems; and intelligent healthcare for detecting Ultraviolet (UV) radiation, monitoring patient conditions, and controlling emergency response vehicles. Since the IoT exchanges massive quantities of essential and sensitive data, the lack of security of those networks, especially involving security breaches or penetration, could lead to severe repercussions economically and endanger human lives [7,8].

In IoT, the information is exchanged and routed among the linked devices through a specially designed network that supports IoT specifications. An example of such networks is Low power and Lossy Networks (LLNs), comprising a wide range of embedded devices, such as sensors and actuators. Those devices are the driving force behind the IoT, since they enable global connections to items that are not connected to the Internet. However, these embedded devices have a low power supply, small memory space, limited computing capabilities, and a short radio range. Hence, to enable communication among those appliances, the Internet Engineering Task Force (IETF) specified IPv6 over Low-Power Wireless Personal Area Networks (6LoWPAN) to serve as an adaptation layer and effectively encapsulate long IPv6 headers in packets of 128 bytes [9,10]. The next section provides more details about the architecture of the RPL protocol.

### 1.1. Routing Protocol for Low Power and Lossy Network (RPL)

Due to the constrained environment of LLN, it is obligatory to conserve the energy of such devices while transmitting and transferring information among networks’ nodes. Therefore, several protocols have been devised and standardized to allow and manage the communication amongst LLN’s resource-constrained devices. One of the most popular proposed protocols for routing purposes in LLNs is RPL [11].

The RPL is a standard routing protocol for LLNs established by the IETF Routing Over Low power and Lossy network (ROLL) task force in 2012 and detailed in Request for Comment (RFC) 6550 [12]. IEEE licensed the development of RPL to overcome the current gap in the routing of IoT networks and attain the limited capabilities of LLN’s devices. The fundamental concept underlying RPL is the topological concept of Destination-Oriented Directed Acyclic Graphs (DODAGs).

The DODAG is a directed graph with no loops oriented towards a root node. The nodes that provide Internet access (gateways) are called root nodes, and the other nodes in the network are linked with it either directly or indirectly through a sequence of parent nodes. Furthermore, each node is responsible for selecting the desired parent, who is then used for forwarding the application packets. The parent node selection depends on the rank value that a device (node) can achieve. Moreover, the rank value refers to the position of a node in the DODAG. Hence, the rank value is affected by the node’s distance from the root and the Objective Function (OF). The OF determines numerous metrics, such as rank of nodes, selection of the parent node, and route optimization. The versatility of RPL to interact with many limited devices is the primary reason for its adoption in LLNs [11].

The RPL adds five new control messages for constructing and maintaining the DODAG and communication routes. The RPL control messages are a specific type of Internet Control Messages Protocol version 6 (ICMPv6) control message, as follows:

- **DODAG Information Solicitation (DIS).** Nodes intending to join a network but have yet to receive DODAG Information Object (DIO message advertise a DIS message to inquire for available DODAG to create a connection).
- **DODAG Information Object (DIO)**. Nodes use the DIO message for locating RPL instances, learning about DODAG configurations, choosing a preferred parent, keeping DODAG structure in place, and knowing the current rank of the node and the IPv6 address of the root [13].
- A **Destination Advertisement Object (DAO)** message is used for advertising backward route information by building upward and downward routes between nodes and then creating routing tables on receiving nodes [14].
- A **Destination Advertisement Object Acknowledgement (DAO-ACK)** message is a response message to a DAO message.
- **Consistency Check (CC).** The RPL protocol employs CC to ensure the synchronization of the “security counter or timestamp between each pair of nodes” [15].

The RPL supports various communication paradigms, Point-to-MultiPoint (P2MP), Point-to-Point (P2P), and MultiPoint-to-Point (MP2P) [15]. In addition, the RPL supports two modes of operation. First, the storing mode, where each node maintains a downward routing table for its sub-DODAG and uses it to transmit P2P traffic. Consequently, the traffic will go upward until it arrives at a common predecessor (of the sender and destination), then it will be forwarded downward to the destination node. Second, the non-storing mode, where the root node is the sole device that retains. Application packets are first sent to the root node, then re-routed to their destination in this mode. Those looking for further explanations of the architecture and implementation of the RPL protocol can find them in [16].

### 1.2. Security Issues and Threats in the RPL Protocol

According to a recent report by Nokia [17], attacks on IoT devices are increasing at an alarming rate. The increase is due to the proliferation of automated tools to exploit IoTs’ vulnerabilities. The report states that IoT devices now make up roughly 33% of exploited devices, compared to only 16% in 2019. The statistics are the outcome of monitoring aggregated network traffic data of more than 150 million devices globally. Furthermore, researchers claim that more than half of all deployed IoT devices are vulnerable to medium to high severity attacks [7].

The exponential increase in the demand for IoT devices has accelerated research and development efforts in IoT-related areas, including security. It has attracted many researchers to investigate attacks targeting IoT networks. Many IoT applications use the RPL protocol, since it is purposely developed for constrained devices commonly used in modern IoT applications. Nonetheless, the RPL protocol is still vulnerable to many threats that could harm the entire network infrastructure. Consequently, researchers invest their time and efforts investigating various threats in LLNs that use the RPL protocol [18].

Routing security is a major concern, as routing-related attacks impede sensor data transmission and adversely affect network layer performance. It can also significantly affect the upper layers of the IoT network, which frequently causes Denial of Service (DoS) attacks [19]. In addition, providing a suitable security mechanism for the routing protocol in the IoT is challenging due to the characteristics inherited from other networks. Furthermore, the packet forwarding process in IoT-constrained devices is influenced by potential security threats, affecting the delivered services to end-users as the performance of the malignant nodes is rapidly increased during the data packet routing process. Eventually, the network’s topology will be disrupted, and the resulting overhead will deplete the nodes’ power resources and eventually break down the whole network [20].

In addition, the RPL security specification allows the protocol to operate in an open or optionally secured mode. In open mode, any node can join the LLN without an authentication key. In a secure mode, a node requires a preinstalled key to join the LLN and an additional authentication key to join as a sensor with routing capability. RPL also provides an optional consistency check feature for protection against replay attacks. Although RPL provides these optional features to address the security of the routing process, most of the security features are implemented only for external attacks owing to the constrained nature of IoT-LLNs. When a malicious node joins the IoT-LLN, RPL has several exploitable vulnerabilities, allowing adversaries to instigate insider attacks that deplete network resources and degrade performance. Thus, the security of RPL is crucial due to its significance in IoT networks [21,22].

**Figure 1:**
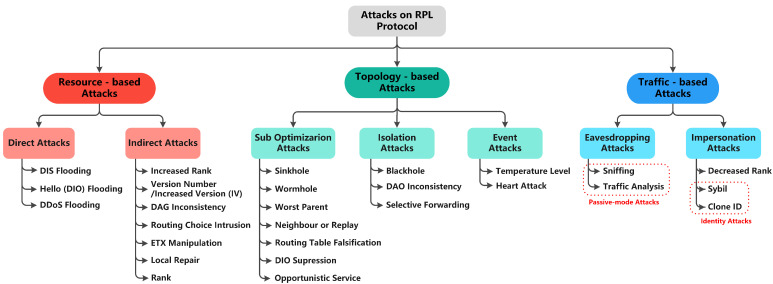
Taxonomy of RPL attacks.

Figure 1 shows the taxonomy of RPL attacks, classified into three categories, and each category has two subclasses based on the intent of threats [23]. These attacks may cause severe network issues, such as exhausting network resources, destructing network topology, and stealing sensitive information. Further, Figure 1 lists the recent attacks addressed by the existing studies. Section 7.6 provides further details about those attacks.

To this end, many researchers have suggested that addressing routing attacks in RPL- based LLNs is still an open research issue [23]. However, due to the constrained and open nature of RPL-supported IoT-LLNs, implementing complex security solutions is difficult, and that leaves the network vulnerable to attacks. Therefore, there is a need for designing an efficient approach/solution to address routing attacks in the early stage of any malicious activity within the network [24].

### 1.3. Machine Learning (ML) and Deep Learning (DL) Technique for RPL Security

The existing traditional security mechanisms, such as those based on cryptography, trust, threshold, and Intrusion Detection systems (IDS), cannot detect or prevent RPL-LLN attacks effectively due to differences in the topology, complexity, and dissimilarity in the traffic patterns [18,20].

In addition, these mechanisms fail to detect sophisticated and devastating huge attack surfaces and require heavy resources, which drastically deplete the resources of constrained devices and impact the normal operation of network nodes [25]. Furthermore, detecting a complex and a combination of multiple attacks in the network requires an intelligent detection mechanism. Hence, there is a need to design a detection mechanism capable of handling the issues described above. The security mechanisms should be eligible to identify known and unknown (zero-day) attacks with a thorough examination of their actions. Additionally, the vast amounts of data traffic exchanged between the sensors (devices) of the LNN-based IoT network constitutes another challenge that should be considered throughout the design of the detection mechanism.

The term “big data” refers to a high volume of data. Such data necessitate advanced techniques to extract valuable information to analyze legal and harmful behavioral packet patterns. "Big Data" is a buzzword that includes methods to extract extremely vital information from the massive amounts of data traffic exchanged in the IoT networks. In other words, not all the data traffic is necessary for further analysis and learning. The advancements in big data technology make it more practical to extract different legitimate and malicious behavioral packet patterns from the immense data traffic [26].

Unfortunately, conventional intrusion detection methods cannot effectively process large amounts of data, resulting in a lack of useful information. Thus, more intelligent and adaptable mechanisms are needed to expand the detection capabilities of the existing security defenses systems for the next generation of IoT networks. ML learning and DL-based intrusion detection techniques have attracted much interest in enhancing security in IoT networks [27]. With its ability to learn from datasets, ML is particularly suited to complexities that are too complex to be fully explained or performed precisely. Furthermore, ML requires a small amount of data for training and testing but has lower accuracy. On the contrary, DL, a subset of ML, requires a vast quantity of data to train the system and takes a long time, but it usually gives higher accuracy [28,29]. In that sense, ML and DL are the most successful computational techniques for providing embedded intelligence in the IoT context due to their ability to deal with a tremendous amount of data, maximize feature engineering, learn from latent abnormal patterns, and reduce the time for detecting known and unknown attacks [30,31,32,33]. Thus, the ML and DL approaches improve the IoT security and RPL network in particular and overcome the weaknesses of other conventional solutions.

Due to the limitations of IoT devices in terms of computing and power resources, designing ML and DL algorithms for the IoT network is a challenging endeavor [34]. In this context, ML and DL techniques are applicable at RPL nodes, fog/edge nodes, and (or) cloud nodes to extract and analyze large-scale data to detect malicious behaviors. As a result, Artificial Intelligence (AI)-assisted security analysis approaches can transform end-to-end IoT security into an intelligence-based monitoring system [35]. Recently, ML- and DL-based security solutions for identifying attacks and countering threats intelligently in the RPL network have become a promising research area and have attracted attention from many researchers to add more to this field.

### 1.4. Contributions and Structure of Study

The contributions of this study are six-fold, as follows:

- Provide a Systematic Literature Review (SLR) for the state-of-the-art approaches concerning ML, DL, and combined ML-DL approaches to detect attacks in RPL-based 6LoWPAN.
- Introduce theoretical and practical steps for conducting SLR studies that pave the way for other researchers to conduct their SLRs in any field of academic research.
- Provide a taxonomy on contemporary research directions in RPL-based 6LoWPAN.
- Demonstrate demographic, statistical, and critical analysis on the existing studies with the implemented attacks and used tools.
- Clear description and analysis of the benchmark datasets created and used by existing studies in the RPL-based research field.
- Derive various security issues and challenges of previous studies and provide future research directions.

The study is structured as follows. Section 2 summarizes relevant works and compares them to ours, while Section 3 presents the research questions and methods. Section 4 provides an overview of the methodology and stages of this study, identifies the databases, highlights the checking and refining criteria. In Section 5, we present the distribution of the final selected studies. Section 6 demonstrates the theoretical and practical steps for conducting the SLR study. In Section 7, we elucidate the result of the study according to the designed research questions in detail. Then, the existing challenges of the presented studies and some possible research directions are in Section 8. Finally, the conclusion and limitations of the study are presented in Section 9.

## 2. Relevant Works

This section presents the existing literature studies within the scope of attack detection in RPL-based 6LoWPAN in IoT networks related to our study. The related works in this section focus on existing reviews, surveys, and systematic review studies. In addition, we identify and highlight the covered topics along with the limitations and gaps of such studies.

In the research of [16], the authors provided a survey on the existing challenges in IPv6 for LLNs. The authors presented a thorough background on the RPL architecture and its network operation; additionally, the authors reviewed 97 papers related to the RPL research domain. The reviewed studies were analyzed and classified into 13 categories according to RPL specifications. In addition, this work listed statistics concerned with practical parts of reviewed studies (e.g., simulation method, number of experiments, number of parameters, and other hardware and software specifications). Furthermore, the authors covered several RPL security threats, albeit partially, and offered some ideas for future research directions.

The authors in [36] provided a review of the security of RPL-based 6LoWPAN in the IoT. First, the authors provided an overview of the IoT architecture and the RPL protocol, then explained the existing threats in RPL with some proposed defense mechanisms. In addition, this work presented the evaluation metrics used in those mechanisms. Finally, based on shortcomings extracted from the existing review studies, the authors discussed the issues and proposed some paths for future research.

The authors of [25] surveyed various solutions for detecting IoT attacks using ML techniques. They presented a thorough background on the IoT layers and security issues, including different attacks targeting IoT layers. Moreover, the authors proposed various classifications for cyber-attacks based on behavior type, targeted layer, and type of damage. They also presented some ML techniques used in attack detection supported by different statistical results. However, they only briefly explained RPL attacks before discussing ML challenges and potential solutions.

In [37], the authors propose an SLR on the security aspects of the RPL protocol. After a detailed introduction to the RPL architecture, the authors reviewed 53 proposals covering different aspects of RPL attack countermeasure mechanisms (e.g., mitigation, authentication, cryptography, network monitoring, secure parent node selections, and others). Moreover, the authors established a set of research questions as a baseline for their work, then answered them sequentially. Moreover, the authors categorized the collected studies into different classes and provided a comprehensive analysis from a statistical point of view; the authors also displayed a list of tables and info-graphics that facilitate the extracted information. Meanwhile, the authors showed some of the existing challenges with possible solutions. However, they did not give any details or critical analysis about the reviewed studies (e.g., proposed architecture, simulation environment, utilized parameters, conducted results, and others).

In [15], the authors performed a survey on ML approaches for detecting IoTs attacks, focusing on the design of IDS based on ML techniques. The authors explained the significant aspects of IDS systems, implementation, and their taxonomy in IoT networks. Moreover, they also discussed several recent IDS approaches for detecting IoT attacks and identified their limitations. Finally, the authors identified some of the open issues and research challenges with future works that could solve outstanding problems in IoT security.

The authors in [38] reviewed the implementations of several IDS strategies in IoT, providing insights into IDS techniques, deployment strategy, validation approach, and datasets. The authors first presented a taxonomy of IoT attacks with detection and security mechanisms, then offered a taxonomy for anomaly-based IDS techniques involved in IoT attack detection. Moreover, the authors presented a theoretical part of supervised and unsupervised learning in IDS, then compared the ML and DL techniques. They also provided an overview of various attacks on the IoT ecosystem. Finally, the authors discussed some challenges IDS faces in various IoT environments.

The authors in [39] presented an SLR on IDSs in RPL-based 6LoWPAN, covering a total of 103 published studies in the domain. They provided comprehensive information about RPL’s architecture and the negative impact of RPL attacks on the network. In addition, the authors provided a critical review of the collected studies and suggested several potential improvements to the existing studies. Furthermore, they also demonstrated an all-inclusive taxonomy and analysis of IDS-based RPL techniques (e.g., monitoring data source, detection strategy, response, monitoring techniques, validation approaches, and others), followed by an exhaustive statistical analysis of the reviewed studies (e.g., research outcomes, adverse effects of RPL attacks, network simulators, evaluation metrics, and others). In addition, the authors present some of the most commonly used datasets in IoT networks, as well as a brief review of RPLs’ datasets. Finally, the authors identified and thoroughly discussed the gaps and suggested several future research directions.

Another SLR study [40] focuses on using ML techniques to detect IoT attacks. The authors proposed six research questions about ML techniques and IoT security, followed by a detailed overview of IoT attacks and their classification according to IoT layers. In addition, the authors provided a critical review of the existing studies. Furthermore, an analysis of the recent datasets used by researchers in the IoT domain was covered. They also identified and discussed the primary security challenges and issues contributing to the vulnerabilities of IoT devices. Finally, the authors epitomized the challenges and gaps in the existing IoT security trends of the reviewed studies.

Table 1 compares our SLR study with existing literature studies to show the uniqueness of our work compared to others in terms of various metrics, such as RPL Architecture, RPL Security Threats, RPL-ML Technique, RPL-Technique, and RPL Datasets. The authors developed these parameters after a thorough examination and review of the existing literature on RPL security. Such a comparison is necessary to comprehend the issues with RPL security to develop more efficient detection mechanisms for RPL-based attacks. Furthermore, it could be a starting point for future researchers working on this topic. This work is compared to eight previous relevant studies in [15,16,25,36,37,38,39,40].

**Summary**: From Table 1, it is clear that many approaches that focus on the IoT in general and RPL specifically have been proposed recently. Some approaches, such as [16,37], provided a comprehensive background on RPL architecture and shed light on the security threats of RPL with brief information about security, threats, and ML techniques in RPL [37]. In addition, others, such as [36,39], address both the RPL architecture and security threats in detail with a brief overview of ML techniques in RPL [36] and RPL datasets [39]. Moreover, other researchers [15,25] partly covered RPL Security and Threats and did not cover other topics such as ML techniques in RPL. In spite of that, the researchers [15] provided an abstract review of the DL technique in RPL. Furthermore, one study [38] slightly covered the RPL architecture and its security threats without any attention to the other topics. Lastly, one of the studies, [40], only provided a glimpse into security, threats, and ML techniques in RPL, and it lacked details on other areas related to the RPL research domain. As a result, some approaches cover the RPL architecture and security threats in detail and lightly cover ML and DL techniques in RPL.

Consequently, there is still a need for a well-planned and designed SLR for aspects not yet covered in other approaches. Therefore, to the best of our knowledge and when writing this study, our proposed study is the first SLR on ML and DL approaches in RPL with an extensive review of the benchmark datasets used to evaluate RPL-based IDS approaches within IoT networks.

## 3. Research Questions and Method

This study aims to investigate state-of-the-art studies, gather their findings, and recapitulate their empirical evidence associated with the application of ML and DL mechanisms for identifying suspicious behaviors in RPL-based 6LoWPAN networks. Furthermore, this study presents the existing researchers-generated datasets and benchmarked datasets. To conduct the aspirated goals, we define sets of research questions as follows:

- **RQ1:** What is the distribution of the selected studies according to the year of publication, digital library, publication type and topic, and country of origin?
- **RQ2:** What are the existing ML-based approaches to detect attacks in RPL-based 6LoWPAN?
- **RQ3:** What are the prevailing DL approaches contributed by existing studies to detect RPL-based 6LoWPAN attacks?
- **RQ4:** What state-of-the-arts combined ML and DL approaches have been used to detect attacks in RPL-based 6LoWPAN?
- **RQ5:** What are the recent applications based on ML and DL approaches proposed for detecting attacks in RPL-based 6LoWPAN?
- **RQ6:** What are the existing threats in RPL-based 6LoWPAN that the existing studies had addressed?
- **RQ7:** What tools and network simulators are used in the existing studies, and what are the occupied evaluation metrics and parameters in the reviewed studies?
- **RQ8:** What are the datasets utilized to evaluate the existing studies, and are there any available datasets designed specifically for RPL-based 6LoWPAN?

Regarding the research method used in this study, we followed the guidelines presented by PRISMA [41].

## 4. Research Methodology

In this section, we explain the methodology of the proposed SLR study in detail and provide a clear description of each stage of the study. To achieve the primary goal of this work, we designed three main stages, and each stage comprises several steps illustrated in the following subsections.

### 4.1. Stage 1—Identification of Information Sources and Research Keywords

This study conducted the search and collection process for relevant studies and articles until June 2021 on three databases and two data sources to extract and collect related studies from the literature. The databases comprise Springer Link, IEEE Xplore^®^ digital library, and Science Direct, and the data sources include Scopus and Google Scholar. The electronic link for databases and data sources searched are as follows:

- Springer Link (http://link.springer.com; accessed on: 1 April 2022).
- IEEE Xplore^®^ Digital Library (http://ieeexplore.ieee.org; accessed on: 1 April 2022).
- Science Direct (http://www.sciencedirect.com; accessed on: 1 April 2022).
- Scopus Database (http://www.scopus.com; accessed on: 27 April 2022).
- Google Scholar (http://scholar.google.com; accessed on: 1 April 2022).

A set of research keywords are identified to narrow down the scope of this research. This study used broad and specific keywords to obtain a reasonable number of studies related to the research topic. The broad keywords represent the IoT field, and the specific keywords refer to the RPL research domain. The research keywords of this SLR were two-fold as follows:

- The first group of keywords includes ((rpl OR “routing protocol” OR “Routing Protocol” OR 6lowpan OR RPL) AND (iot OR “internet of thing” OR “Internet of Things” OR IoT)) for retrieving the studies from (Springer Link, IEEE Xplore^®^ digital library, Science Direct, and Scopus).
- The second group of keywords comprises ((rpl or “routing protocol”) AND (iot OR “Internet of Things”)) for extracting studies from the Google Scholar website, since we observed that Google Scholar returned too many results when using the first groups of keywords. Therefore, to solve this issue, we eliminate some of the keywords not directly related to our study scope, resulting in a more manageable number of gathered documents.

**Figure 2:**
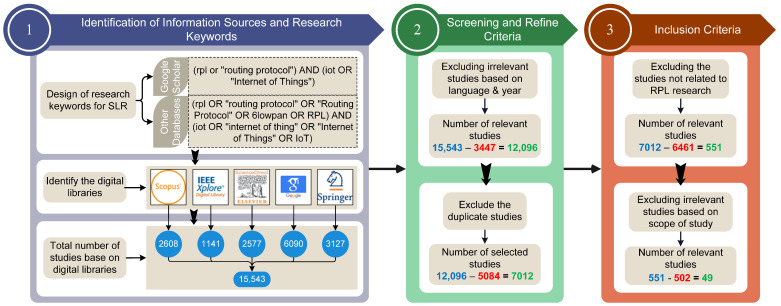
Flowchart of the SLR methodology stages.

In addition, we considered the upper and lower case of keywords. Figure 2 shows the stages of the SLR methodology.

### 4.2. Stage 2—Screening and Refine Criteria

This stage explains the exclusion criteria used in this study. First, we only consider the articles published between January 2016 to mid-2021. Second, we limit the articles in our study to those published in the English language by using each database’s language selection option. However, since Google Scholar, Science Direct, and IEEE Xplore^®^ digital library do not provide a language selection option, we collected all relevant studies from those sources. Next, we selected English-only studies by screening the title and abstract using Mendeley Software [42]. We discovered that several studies were in multiple databases during the screening process. Therefore, we started removing the duplicated studies using Mendeley; additionally, we performed another filter to check the type of documents. This procedure limits the document types to journal articles, conference proceedings, and book section articles and excludes others, such as dissertations, reports, presentations, and magazines.

### 4.3. Stage 3—Inclusion Criteria

In this stage, we screened the titles and abstracts of the articles in Mendeley Software to select the studies related to RPL research and exclude unrelated areas, such as Wireless Sensor Network (WSN) and ad hoc networks. Then, we performed a full-text reading to select the studies within the scope of our study. Consequently, we included only the studies that used ML and DL mechanisms for detecting attacks in RPL networks and studies that utilized benchmark datasets in RPL networks. Last but not least, we have to exclude inaccessible studies due to limited access to the databases.

## 5. Distribution Results of SLR Stages

The selection of studies for SLR was made in three stages, as shown in Figure 2. Applying the research keywords on five digital libraries in the first stage returned 15,543 results, where 2608 are from Scopus, 1141 from IEEE Xplore^®^ digital library, 2577 from Science Direct, 6090 from Google Scholar, and 3127 from Springer. Filtering the results between January 2016 and mid-2021 and the English language option in the second stage reduces the returned results to 12,096 studies.

**Figure 3:**
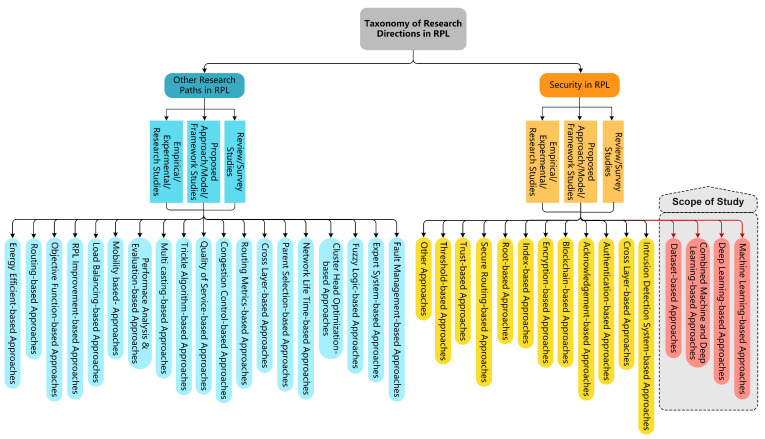
Taxonomy of existing research literature in RPL.

However, approximately 5084 duplicate studies were discovered, reducing the total relevant studies to 7012. Later, in the third stage, filtering for the RPL research topic, a further 6461 studies were excluded, resulting in 551 studies. Meanwhile, from the selected studies, we provide a novel taxonomy of existing research literature on RPL, as shown in Figure 3. Such a taxonomy aids researchers by providing state-of-the-art research directions for the RPL protocol. Finally, we performed a full-text reading on RPL-based studies to extract the studies related to the scope of this study, resulting in a final count of 49 studies.

## 6. Theoretical and Practical Steps for the SLR Study

This section presents and explains the steps involved to conduct the SLR study in detail, including the tools, software, and techniques used. The process is replicable in other SLR studies in any field of academic research. The following are the practical steps undertaken for each database.

**Figure 4:**
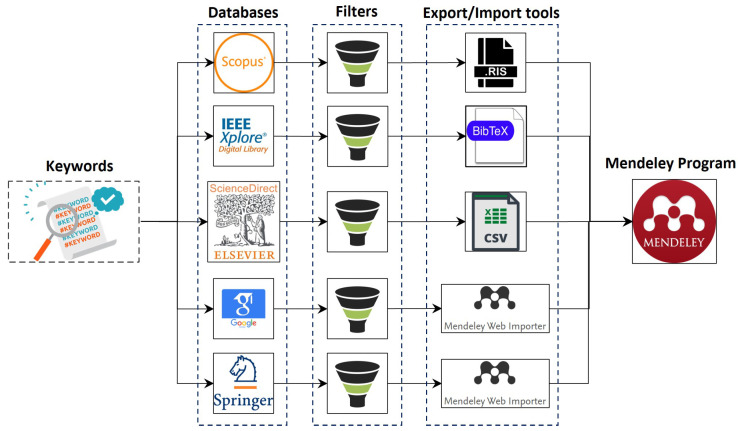
The steps and tools for conducting the SLR study.

- **Google Scholar:** First, we inserted the second group of keywords (G.2) into the search box; then, we selected the range of publication years, resulting in the required documents displayed and available to us. However, we had to exclude several duplicate documents found in other databases when using the first group of keywords throughout the search process. Then, we imported the residue documents into the My Library feature in Google Scholar before exporting them to Mendeley Software using the Mendeley Web Importer Tool’s extension [43].
- **Scopus Database:** The first group of keywords (G.1) is inserted into the search box. Then, the search results are refined using year and document type filters. Afterward, we exported the selected studies using the Research Information System (RIS) before importing them into Mendeley software for further analysis.
- **Springer Link:** We began by entering the first group of keywords (G.1) into the search engine, then refined the returned results based on document type and year of publication. Then, we downloaded the links to the selected articles as separate CSV-formatted files. Next, we downloaded the articles from the available links and imported them into Mendeley software using Mendeley’s Web Importer Tool extension [43].
- **IEEE Xplore^®^ digital library:** We began by inserting the research keywords (G.1) into the search box, then applied the filter and selection criteria. Then, we export the selected documents using (.bib) format, which is then imported into Mendeley Software.
- **Science Direct:** The search process in Science Direct is similar to that for the Scopus database. We selected the displayed documents according to the pre-defined criteria. Then, we exported the documents in RIS format, which can be imported into Mendeley Software later. Figure 4 shows the whole process of conducting the SLR stages.

## 7. Result and Discussion

In this section, we explain the results of the SLR study and provide answers to the research questions based on the findings of the selected studies.

### 7.1. RQ1: What Is the Distribution of the Selected Studies According to the Year of Publication, Digital Library, Publication Type and Topic, and Country of Origin?

**Figure 5:**
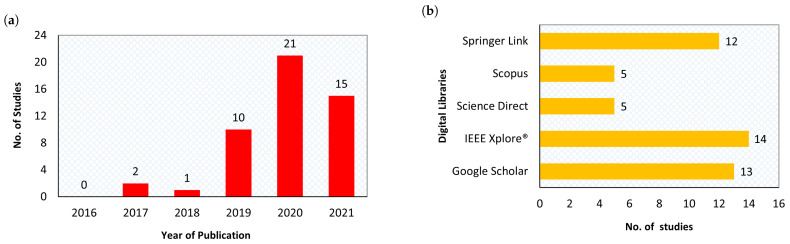
Distribution of selected studies according to (**a**) Year of Publication, (**b**) Digital Libraries.

We answer this question by providing a list of figures and charts that describe the bibliographic information of the selected studies. Figure 5a depicts the distribution of selected studies according to the year of publication, and Figure 5b shows the allocation of selected studies in the digital libraries. We can observe from Figure 5a that the publication related to the topic of this study is constantly increasing over the years. Looking at the chart, we concluded that the number of published studies in 2020 almost doubled from the year before. After conducting this study in June 2021, we predict a marked increase in the number of studies on ML and DL on RPL published in 2022, indicating the significant role of ML and DL mechanisms in identifying attacks in RPL-based 6LoWPAN at present and in the future.

Figure 5b shows that most related studies were published in IEEE Xplore^®^ digital library (14), followed by Google Scholar (13) and Springer Link (12), while Scopus and Science Direct returned the same number of published studies (5). Furthermore, it is worth mentioning that there are many studies published by other publishers, such as MDPI and Wiley, but rather than directly extracting them from the publishers’ databases, we obtained those studies via the Google Scholar database.

**Figure 6:**
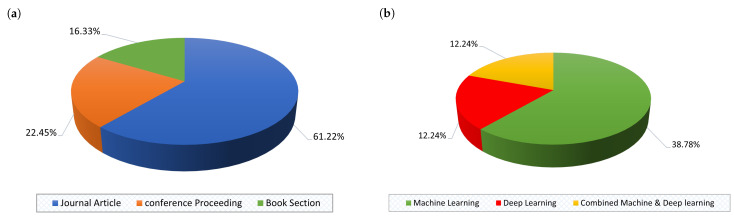
Distribution of selected studies according to (**a**) Publication Per Type, (**b**) Publication Per Topic.

Figure 6a,b show the percentage of the selected studies according to the type and topic, respectively. As for the type of published studies, it is evident that most were journal articles, i.e., 30 out of 49 studies (61.22%), while 11 were indexed conference proceedings (22.45%), and only 8 (16.33%) of the studies belong to the book section. Furthermore, regarding the topic of selected studies, it is evident that many published studies emphasize ML mechanisms, which constitute 23/49 (46.94%) of published studies, while 6/49 (12.24%) of studies focus on DL mechanisms, and the last portion, 6/49 studies (12.24%), focus on combined ML and DL mechanisms. In addition, it is worth pointing out that the studies that provide a review on the topic, 8/49 (16.34%) (see Section 2), and the studies that proposed a mechanism without empirical results, 6/49 (12.24%), are not presented with the distribution of studies in Figure 6b.

**Figure 7:**
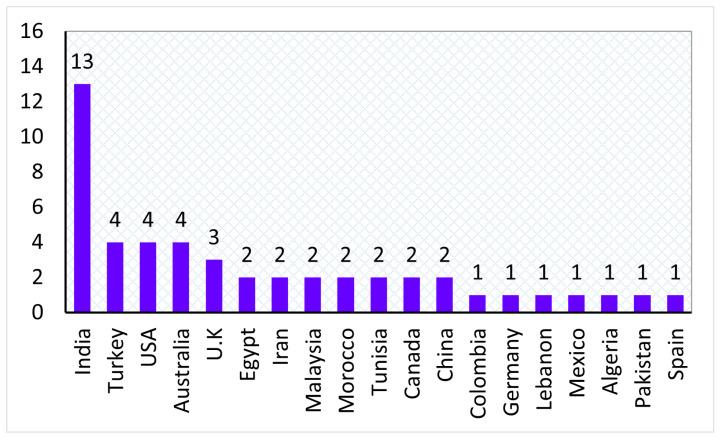
Distribution of studies according to country of origin.

Figure 7 shows the total number of selected studies based on country of origin. As can be observed from the chart, India is the most active country in this field of research, followed by Turkey, USA, Australia, etc.

### 7.2. RQ2: What Are the Existing ML-Based Approaches to Detect Attacks in RPL-Based 6LoWPAN?

This section produces a classification of the reviewed studies based on the ML mechanisms described in Section 7.1. Then, we illustrate the most crucial information from each study. After that, we figure out a summarization table that highlights the crucial parameters with the limitations of existing studies.

Shukla [8] proposed an ML model with lightweight IDS to detect WormHole (WH) attacks in IoT networks. The proposed approach contains three ML models: K-Means (KM) clustering-based IDS (KM-IDS), Decision Tree (DT)-based IDS (DT-IDS), and a two-stage hybrid IDS approach (KM Clustering and DT models). The first model used centralized IDS to identify attacks by sorting network nodes into clusters. Each cluster, called “safe zone” plays a crucial role in identifying attacks inside the network, while the second model also used a centralized IDS; this model is used to train the data that help to specify the safe distance between two neighboring routers, which is later used as a baseline for identifying attacks. Then, the IDS is used to detect the attack; for example, if two routers send a request to become neighbors, the IDS will check if the safe distance between the two routers is within the specified safe distance. This decision is created through the learning stages to check whether the two neighbors’ routers are normal or victims of the attack.

Eventually, the last model uses the KM clustering algorithm to set up a safe zone to detect WH attacks. After that, the DT algorithm reduces the occurrence of false alarms and improves the attack Detection Rate (DR). The author used two types of networks in the experiments. The first network comprises randomly distributed network nodes, while the second network represents standard topologies, such as mesh, ring, and star. The simulation result shows that the KM approach obtains 70–93% DR for different sizes of random RPL networks, while the second model, DT-IDS, achieves 71–80% DR. Additionally, the hybrid model attains 71–75% DR for the same network size. The result clearly shows that the hybrid model achieved the lowest result compared to the others. However, the hybrid model has significantly reduced the False Alarm Rate (FAR), while the other two models still suffer from high FAR.

The study [44] presented an IDS based on a Self-Organizing Map (SOM) neural network that clusters the WSN routing attacks and reports them to the network administrator if an attack is detected. This work targeted three types of attacks: HF, SH, and VN. In addition, the authors used the Cooja simulator to create a synthetic dataset. During the simulation, the authors compute the PRC of every node utilizing Contiki’s Power Tracker module. Then, the data are fed to an aggregator to perform some processing and remove unnecessary information from the packet. Finally, the processed data are inserted into the train to obtain the SOM map. Moreover, the authors assigned specific types of SOM parameters, for example, the Euclidean distance for the distance function, a Gaussian function as a neighborhood function, and a random initialization of the SOM weights.

Furthermore, the authors extracted six types of dataset features using the available data in packet fields. The time window technique was employed as a reference to identify suspicious behavior at a particular time slot. As for the simulation result, the authors reported that the SOM model could accurately cluster the dataset samples into four classes: clean data, HF, VN, and SH attacks.

Anitha and Arockiam [45] conducted an Artificial Neural Network-based IDS (ANNIDS) technique for detecting attacks in RPL. The proposed approach uses MLP to identify DIS and VN attacks. The architecture of the proposed approach contains three phases; the simulation phase for generating and collecting the real-time data packets, preprocessing phase to extract the features from network packets, and the last phase, which is ANNIDS. The ANNIDS phase uses the MLP classifier to identify the normal and malicious traffic. In addition, the authors set up the simulation using a random distribution of network nodes with two attacker nodes. The results reveal that the Mean Absolute Error (MAE) is 0.0002 and the Root Mean Square Error (RMSE) is 0.0003. Meanwhile, the other evaluation metrics, such as TPR, Precision, Recall, and F-Measure, attained the maximum result in this experiment.

The authors in [46] conducted an ensemble learning approach based on Network IDS for detecting attacks in RPL-based IoT networks. The proposed approach, named ELNIDS, is designed to detect seven types of routing attacks (SinkHole (SH), BlackHole (BH), Sybil, Clone ID (CID), Selective Forwarding (SF), Hello Flooding (HF), and Local Repair). The architecture of the ELNIDS approach consists of several modules: sniffer for listening to network traffic, a repository for archiving the sensor events, a feature extraction module, an analysis module, a database for signatures, a user interface, and a notification manager for attack alerting. The working principle of the ELNIDS approach is based on ensemble learning that combines different kinds of ML classifiers. In this work, the authors used four types of ML classifiers: Boosted Trees, Subspace Discriminant, RUSBoosted Tree, and Bagged Trees.

The authors evaluated the performance of each classifier individually using different evaluation and validation metrics. Then, the ensemble model (voting scheme) is applied to improve the classification results by merging multiple models. The output of this method produces better prediction Accuracy (AC) and generates less noise than the traditional single ML methods.

To test the performance of the proposed approach, the authors used their self-generated dataset, RPL-NIDDS, which comprises seven types of routing attacks with twenty features. The authors used two evaluation and validation methods, hold-out and cross-validation, to obtain the experimental results. The simulation results show that the ensemble of Boosted Tree obtained the best AC at 94.5%, with an Area Under Curve (AUC) of 0.98, using 30% and 40% hold-out validations. However, the Subspace Discriminant model obtained the worst AC (77.8%), with an AUC of 0.87, in the case of 40% hold-out. As for the cross-validation method, the ensemble model of Boosted Trees attains the best AC. Meanwhile, the ensemble of the RUSBoosted model produced the highest value of AUC.

Sharma et al. [47] proposed a new framework for simulating attacks in the RPL network by generating a multi-class dataset for a supervised ML model. The proposed dataset consists of 58 features extracted from the networks’ packets gathered throughout the simulation. In addition, the dataset contains the traffic pattern of a regular network and various RPL attacks, such as HF, DIS Flooding, Version Number (VN), and Decreased Rank (DRA) attacks. The authors used pairwise correlation, a dimensionality reduction method, that eliminates variables that have more than a specific correlation among themselves. Moreover, the authors used a correlation ranking filter to remove irrelevant features that might mislead the detection algorithm during the feature ranking and selection stage. The feature ranking technique works by measuring the Pearson’s correlation between the dataset’s features and the class value.

Moreover, the feature ranking and selection stages’ outputs are four datasets with various features. Then, the produced datasets are evaluated separately using three types of classification algorithms: Random Forest (RF), Naive Bayes (NB), and J48 classifiers. After the evaluation process, the authors observed that the dataset with 21 features obtained the highest classification AC. Furthermore, the experimental results show that the RF classifier obtains the best Precision (99.4%), Recall (99.3%), and AC (99.33%) than other classifiers. In contrast, the J48 classifier attained the worst results in all evaluation metrics compared to other classifiers. Finally, the results also show that the dimensionality reduction step significantly reduces the processing time and energy consumption and improves the overall performance of the proposed framework.

A study by [48] presented a centralized IDS for detecting HF and VN attacks in RPL-based Industrial IoTs (IIoTs). The proposed framework uses Genetic Programming (GP) with a centralized IDS to identify the attacks. The proposed mechanisms are placed in the root node to monitor the packets of the surrounding nodes. Then, the monitored packets are analyzed to extract the features that contribute to detecting the attacks. In addition, implementing centralized IDS in this work reduces the computation and communication overhead on the monitoring nodes. The authors used different time intervals (500 ms, 1000 ms, 2000 ms, 3000 ms, 4000 ms, and 5000 ms) in the feature collection step.

Furthermore, the authors examined the distributed IDS type; this type allows the monitoring nodes to identify the attacks. The simulation results show that the proposed framework (Centralised IDS) achieves 96.08% and 99.83% of the worst and best AC values obtained during 500 ms and 5000 ms intervals for the HF attack scenario. For the VN attack, the best and worst AC occurred at intervals of 4000 ms (99.42%) and 3000 ms (97.97%), respectively. As for the distributed architecture, the authors discovered through the experiments that about 51% and 71% of nodes could detect HF and VN attacks, respectively, with an AC rate higher than 90.

An analytical study on the performance of IDS for RPL-based IoT networks is proposed by [49]. The proposed work presents the statistical analysis of RPL-NIDDS17. The authors study the probability distribution of the dataset features and their correlation by applying five types of ML techniques to evaluate the dataset. The workflow of the analysis comprises three steps. Firstly, the authors performed descriptive statistical analysis using three methods: the Kolmogorov–Smirnov test, skewness, and kurtosis functions. Secondly, the authors tested the feature correlation of the training and testing sets by applying Pearson’s Correlation Coefficient (PCC) and Gain Ratio. The process of correlation analysis has two categories. First, the authors used PCC to study the feature correlation of the Training set and Testing set without depending on class or labels. Then, the correlation of features is ranked using the Gain Ratio technique, considering the instance labels.

Lastly, in the third step, the authors evaluated the complexity analysis of the training and testing sets using five ML classifiers: NB, DT, Logistic Regression (LR), Artificial Neural Networks (ANN), and Expectation-Maximization (EM) Analysis Clustering. Afterward, they evaluated the classifiers’ performance using AC and FAR metrics. Additionally, the results of the RPL-NIDDS17 dataset are compared with some datasets typically used in traditional networks, such as KDD-99, UNSW-NB15, and WSN-DS. The authors reported that the DT achieves the best AC (93%) and a minimum of 3.57% for FAR. However, the EM clustering has the lowest AC (89.63%), with a FAR of 5.59%. Finally, the EM and LR classifiers achieve unacceptable results for FAR, and the authors claim this is due to some unbalanced distribution of dataset records.

A study by Neerugatti and Reddy [50] presented an ML approach named MLTKNN for detecting Rank attacks in RPL-based IoT networks. The proposed technique uses the K-Nearest Neighbor (KNN) algorithm to identify the attacks. The proposed approach is placed at the border router node to check the distance of each node physically within the radio range from node to node. The detection process of an attacker node depends on the distance between nodes in the network and the border node. Then, the computed distance is compared to the actual rank of the inspected node. If the two values are not equal, the inspected node is verified as malicious and eliminated from DODAG. Otherwise, it will be considered a regular node.

The authors claimed that the proposed approach achieves better results than the network under rank attack only based on the experimental results. The proposed approach reduced the End to End (E2E) delay from (2.7 to 1.9) seconds compared to the network scenario under attacks when the number of nodes involved is five. Moreover, the Packet Delivery Ratio (PDR) also improved from 59% to 80% after applying the proposed mechanism in a scenario involving 15 network nodes. Furthermore, for the True Positive Rate (TPR), the proposed approach achieved 90% and 98% TPR when the number of network nodes is 5 and 30, respectively. Finally, for the False Negative Rate (FNR), the proposed approach obtained 0.9% and 0.2% scores when there are 5 and 30 network nodes involved, respectively.

Research conducted by Műller et al. [51] presented a distributed anomaly detection of a single mote attack in RPL networks, targeting three types of RPL attacks: HF, VN, and BH. The proposed approach deploys a pre-trained model in the network nodes to avoid extra steps, such as data collection and model training. Furthermore, the authors used a distributed architecture to eliminate communication overhead. Moreover, the proposed approach was evaluated using a semi-supervised ML algorithm suitable for a resource-constrained environment and produces a low computational overhead.

The authors use Kernel Density Estimation (KDE) for the anomaly detection system due to its advantages, such as its ability to provide some degree of prolific illustration and its adaptability to work in highly resource-constrained environments. However, despite KDE providing many benefits, it still has limitations, prompting the authors to modify their model to overcome those limitations. The workflow of the detection phase contains two stages performed at node-level and root-level; the former for anomaly scoring and the latter for anomaly notification packets. Referring to the implementation part of this work, the authors used a modified version of the RPL attacks framework. They initially created 20 test groups containing 80 individual datasets, and each dataset comprises two subdatasets: a normal scenario and a malicious scenario. As for the result of the proposed approach, the authors reported that the system, on average, detects the VN, HF, and BH attacks with (96.1%, 90.2%, and 68.52%) of TPR, respectively, and 0.5% False Positive Rate (FPR). As for the model overhead, the result shows that the proposed approach increases the executable size by 17%.

A Feedforward Neural Network (FNN) model for detecting suboptimal path attacks in RPL was devised by [24] to detect Worst Parent (WP) attacks and the attack sources. First, the authors defined a threat model to analyze the performance of the RPL network under WP attacks. Then, they registered some routing metrics that indicate the presence of WP attacks. In addition, the methodology of the proposed work consists of several stages: data collection, data preprocessing, FNN model initialization, and performance evaluation. In the data collection stage, several steps are used for transforming, encoding, splitting, and scaling collected data. The data are then fed into the FNN model before constructing it and setting up the required parameters.

The designed FNN model identified WP attacks with 18 nodes in the input layer and two hidden layers with one output layer in the performance evaluation stage. Furthermore, the authors assigned Rectified Linear Unit (ReLU) as an activation function and specified a sigmoid activation function for the output layer. Furthermore, the authors employed an adaptive learning rate optimization algorithm to train the FNN model. Concerning the experimental results, the authors reported that they attained the best outcomes of 97.3%, 96.1%, 98%, and 99.1% of AC, Precision, Recall, and F-Score, respectively, with an ELLIPS distribution of 20 nodes in the network.

Canbalaban and Sen [3] presented a cross-layer IDS for RPL-based IoTs. The proposed approach employed a neural network for detecting VN, WP, and HF attacks conducted in binary and multi-class classification scenarios. In this work, the author utilized two types of features extracted from different IoTs’ layers to identify the attacks: routing layer and link-layer features. In addition, the authors introduced and shared their dataset publicly. Such a dataset includes the invoked attacks with different scenarios. Additionally, the authors also came up with a new set of features extracted from the link layer and investigated their effect on intrusion detection. Moreover, the authors classified the extracted features into three groups: data, topology, and link-layer features.

Data-based features include information related to the data packets received by the root node, whereas topology-based features contain information about RPL control messages, and lastly, the link-layer features provide information about the dropped packets in this layer, such information including neighbor allocation, collisions, and queuing.

As for the experimental result for the binary classification model, the 10-fold cross-validation techniques obtained 97.11% and 0.34% of DR and FPR, respectively. In contrast, the 60% split techniques attained 96.88% and 0.13% for DR and FPR, respectively. Moreover, for the multi-class model, the proposed approach achieved a high DR of 97.52%. Furthermore, the results showed that the implementation of link-layer features reduced the FPR and improved the detection of VN attacks.

Qureshi et al. [52] proposed a secure framework for detecting attacks in smart city IoT and Industrial Internet of Things (SFIIoT) in two phases: threshold modulation and attack detection. At first, after identifying all available network traffic features, the features are reduced using GP algorithm. After that, the features with the highest probabilities are elected. Finally, the in-order traversal-based selected features are used to construct a threshold statement for each type of referred attack.

Moreover, after forming the threshold statements, the latter phase identifies the attack. Each attack’s scenario contains a set of features that reflect a specific type of attack. Thus, the targeted attack will be detectable by comparing each scenario with the threshold statement constructed in the previous phase. As for the simulation part of this work, the proposed framework, called RPL-NSF-IIoT, targets specific types of attacks (HF, VN, SH, and BH attacks). In addition, the authors tested the performance of their work using various time intervals (500 s, 1000 s, the 1500 s, and 2000 s) with some evaluation metrics. Based on the simulation result, the authors reported that they achieved the best detection AC of HF and VN attacks at 2000 s, while for SH and BH attacks, the highest result was achievable at 1000 s. In terms of TPR, they achieved the best results at 2000 s for all attacks. Moreover, the experimental results reveal that the PDR of this work is higher, with fewer data drops and delays as well.

The authors in [10] proposed an efficient framework for detecting VN attacks in IoTs that is deployable at the IoT-LLN edge and the cloud. Furthermore, the proposed framework aims to detect the attacks without any miss-identification using any form of deployment. In addition, the detection process in the cloud is performed by a cloud service, while fog computing is responsible for detecting attacks at the IoT-LLN edge. The proposed framework identifies attacks on the local network and cloud side through several stages: filtration of input features, feature preprocessing, and ML classification algorithms (DT, Support Vector Machine (SVM), and Bernoulli RBM and LR).

Moreover, identifying VN attacks in the network relies on various parameters, such as variance in VN and the number of VN changes. Moreover, after identifying malicious nodes in the network, an alert will be sent to the root node to blacklist them. Based on the simulation result, the proposed framework achieves 0.98, 1.00, and 1.00 for AC, Precision, and Specificity, respectively. However, concerning the Recall result, the DT and Bernoulli RBM and LR obtain 95%, while SVM obtains 94

A study by [21] devised an AI technique for detecting SF attacks in RPL. The proposed approach employs a new scheme called the AI-based Packet Drop Ratio (AIPDR), which uses neighborhood information to get the PDR. In addition, the border router node and the other nodes are working mutually based on the environmental situations in the RPL network. The working principle in this work is that all the nodes are checked for the PDR value. Meanwhile, the border router node also inspects the PDR of the other nodes. Then, based on the PDR value, the malicious mode will be detected and eliminated from the RPL network. As for the simulation results, it can be observed that after applying the proposed scheme, the PDR increased up to 89% when the number of nodes was 25. Moreover, when the number of nodes is 25, the E2E result declined from 14 to 4 after launching the proposed scheme. Concerning TPR and FPR, the proposed scheme reports 72% (highest is best) for TPR with 25 networks’ nodes, and the FPR obtained 0.3% (lowest is best) with 25 of networks’ nodes.

In [53], the authosr proposed an ML approach for detecting Rank attacks in a smart hospital environment using centralized anomaly-based IDS. The key specifications of the proposed work include adaptation, lightweight, and learning from the past. For example, the authors selected the One-class Support Vector Machine (O-SVM) for its low Power Consumption (PRC) among ML classifiers for the classification stage. As for the simulation environment, the authors designed four scenarios with a random distribution of malicious nodes and tested their approach with various numbers of attackers’ nodes. The first scenario contains only normal network traffic behavior as training data for IDS, and the other three scenarios have different combinations of malicious nodes. The experimental result shows that the proposed IDS’ DR increases when more malicious nodes are present in the network.

Kumar et al. [54] proposed a DT-Based IDS for preventing intra and inter-network from Denial-of-Service (DoS) attacks. The proposed work investigates the behavior and impact of two types of Distributed DoS (DDoS) attacks: HF and VN attacks. In addition, they used the Cooja simulator to generate their dataset. Moreover, the authors deployed a distributed-IDS on some high-power sensor nodes to prevent intra- and inter-network attacks. Furthermore, the architecture of the proposed approach encompasses several IoT sensors, two of which are for IDS monitoring, and one is responsible for performing actions, which is the border router node. The proposed C5 DT-based IDS model attained 99.9% and 5.2% of AC and FAR, respectively, based on the experimental result. However, it suffered from massive Central Processing Unit (CPU) power and listening PRC under attack simulation compared to normal behavior in terms of PRC.

Tabari and Mataji [55] proposed an anomaly-based distributed IDS for detecting SH attacks in RPL. In this work, a hybrid placement strategy was employed for monitoring and identifying suspicious behaviors. The monitoring nodes were deployed in the host, and the IDS agent responsible for computations and actions runs inside the border router. The workflow of the proposed approach has several stages. First, the data collection stage uses a lightweight module inside the network node to send information to the border router. After collecting the information, the preprocessing step identifies invalid and faulty data using specific techniques. After that, an ML algorithm, GP, selects the significant features that contribute to high classification AC. The classification stage uses three algorithms: DT, SVM, and Bayesian Classifiers.

Furthermore, during the evaluation step, the performance of each classifier was tested individually. Moreover, to enhance AC and reduce the computational cost of alarm processing, the authors propose a post-processing stage that depends on a predefined threshold. Such a threshold was used to revise and validate the generated alarms.

The authors conducted the experiments at different intervals of the network’s run-time. The Bayesian classifier obtained the highest DR at all time intervals. As for the FPR, the DT classifier attained the lowest results at runtimes of 2, 4, and 6 minutes. On the other hand, at 8 and 10 minutes, the SVM classifier gained the best FPR compared to other classifiers. After the post-processing stage, the Bayesian model’s DR improved to 99.03% at ten intervals, and the DT’s FPR reduced to zero at 2-, 4-, and 6-minute intervals of the runtimes, while at 8- and 10-minute intervals, the DT classifier obtained 0.19% and 0.16% of FPR, respectively.

In [56], the authors presented an ML approach for detecting routing attacks in RPL. The proposed approach simulates three routing attacks: HF, DRA, and VN attacks. The authors used ANN to detect those attacks in their work. The workflow of the proposed ANN-based IDS involves several processes: setting up the network scenarios, inspecting the network behavior in the presence of attacks, sniffing, collecting and processing the gathered information, analyzing and classifying the network traffic using ANN, improving the ANN performance via parameter tuning technique, and testing the proposed ANN via hold-out and k-fold cross-validation methods. As for the simulation scenarios, the authors employed four scenarios, with each scenario representing one type of attack, except for the last scenario, which combines all attacks simultaneously.

Moreover, concerning the packet generation in the network, the malicious node produced the maximum number of packets during HF attacks. On top of that, the malicious node also induced the adjacent nodes to generate more packets inside the network during VN attacks. In contrast, the malicious nodes initiated the lowest number of packets during DRA attack scenarios. Moreover, the authors also compared the performance of hold-out and k-fold cross-validation, and they claimed that the hold-out methods required fewer epochs to obtain 100% AC. Additionally, the authors used ten-fold cross-validation methods to avoid the over-fitting problem. Finally, the ANN model achieves an AC of 100% with an optimized value of its hyper parameters.

The authors in [57] proposed a Multi-Layer (MLRPL) model by employing an ANN approach for detecting DRA attacks in RPL. The MLRPL comprises three phases: data preprocessing, feature extraction based, and ANN-based attack detection model. The authors utilized the IRAD dataset, which contains three types of attacks, VN, DRA, and HF, to evaluate their work. The authors merged two datasets (VN and DRA attack datasets) into one named RPL attack dataset during the data preprocessing phase. The produced dataset includes 18 features. In the second phase, the authors used an entropy algorithm known as information gain to evaluate each feature of the entire dataset and an RF classifier to train the entire dataset. The output of this phase is the optimum set of features, which includes eight features, and such features will be inserted into the last phase for attack detection.

Furthermore, different detection scenarios, such as binary and multi-class classification, are employed to test the proposed model. Regarding the experimental results for the binary class, the training and testing AC was 97.14% and 97.01%, respectively. In contrast, for a multi-class model, the training and testing AC is 96.59% and 96.39%, respectively. The overall results of the proposed model revealed that the proposed model obtained significant results of (97.14%, 97.03%, 0.36%, and 98%) in AC, Precision, FPR, and AUC-Receiver Operating Characteristic Curve (AUC-ROC) scores, respectively. Furthermore, the proposed MLRPL model gained better results than [49,58] approaches. Finally, it is worth mentioning that the proposed model is efficient in terms of training time and complexity of the ANN model.

Osman et al. [31] proposed a lightweight ML approach for detecting VN attacks in RPL-based IoT networks. The proposed approach, named ML-LGBM, used Gradient Boosting Machine for detecting the attacks. The methodology of the proposed approach implicates the production of an extensive dataset of VN attack, feature extraction module, LGBM-based classification algorithm, and optimization of the model parameters. Furthermore, the author employed several stages to reach the desired result to detect the attack mentioned above. Such stages encompass design of the RPL network, data collection, data preprocessing, feature selection, and the ML model.

Moreover, the authors store the collected data using different readable file formats in the data collection stage. After that, the data preprocessing stage prepares the data for the feature selection stage, which involves a Forward Feature Selection (SFS) method to extract the optimal subset of features. The outcome of this stage resulted in 11 features selected from 17 features. After that, the selected features from the previous stage are classified using ML-LGBM to detect the normal and malicious behavior. The results show that the ML-LGBM model attained 99.6%, 99%, 99.6%, 99.3%, and 0.0093 of AC, precision, F-Score, True Negative Rate (TNR), and FNR, respectively. Furthermore, the author compared the performance of their approach in terms of (Training Time, Testing Time, Model Size, AC, Precision, FPR, and F-score) with several existing studies, and the proposed model outperformed other approaches. Additionally, as for the consumption of the system’s resources, the model achieved 140.217 seconds execution time and 347,530 bytes of memory size.

An ML approach for securing RPL routing (MLRP) protocol was introduced by [59]. In this work. the authors targeted several RPL-based attacks, namely VN, Rank, and DoS attacks. The MLRP approach comprises two stages: Data Generation and Attack Detection. The former stage focuses on generating the dataset, which involves creating normal and attack scenarios, while the latter stage, called ML with SVM classifier, includes several modules, namely feature extraction, feature selection, labeling, and attack detection.

In addition, during the feature extraction module, the authors extracted the features from PCAP files and applied some preprocessing steps to the collected features. After that, the authors used Principal Component Analysis (PCA) to reduce the number of features and select significant features that contribute to attack detection. Finally, the classification module used SVM to identify and classify RPL attacks based on the training step. The experimental results show that the average AC obtained by MLPR is between 0.90 and 0.92. Moreover, the Recall metric gains between 0.96 and 0.98. Furthermore, the proposed approach reveals 76.8% of PDR compared to other existing RPL-based approaches when using 1474 control messages with 30 network nodes.

Abapour et al. [60] carried out a routing method for the security of RPL networks using the Ant Colony Optimization (ACO) algorithm. The proposed method dealt with the destination nodes as the root node and used the ACO algorithm to improve the security and reliability of the routing process. In this work, the authors deployed the ant of the ACO algorithm at regular intervals in each node to find the best path to the destination node. After that, each node ant finds the next step using the probability formula. Then, the node selects the route with the highest probability. The results show that their method improves the throughput of the proposed protocol compared to SecTrust-RPL [61]. In addition, when the number of nodes increases, the proposed method reduces the number of expected transfers and enhances the network’s throughput, which is also higher than the SecTrust-RPL protocol.

An extension of [53] is presented in this work. The authors [7] proposed an efficient anomaly detection approach for identifying attacks in smart hospitals’ IoT systems. The proposed approach aims to detect two categories of attacks: e-health-related data attacks and IoT network attacks. The former includes events and health attacks, such as manipulations in temperature, whereas the latter involves unusual variations in heartbeat rates. In contrast, the targeted IoT attacks in this work include Flooding, VN, and Rank attacks. Moreover, an SVM classifier was used for detecting the attacks. The authors used SVM with two kinds of datasets, e-health data and network infrastructure data. Furthermore, in this work, the proposed architecture was validated using a prototype and tested with various scenarios regarding e-health, environment, and network intrusion. In addition, the Cooja network simulator was used to measure the scalability of the proposed approach. As for the experimental result, the SVM model obtained a detection AC of 93.4%, 60.8%, and 91.6% for Rank, Flooding, and VN attacks, respectively. Moreover, for detecting event attacks, the authors reported detection AC of 85.7% and 82% of increasing temperature levels and heart attacks, respectively. Table 2 presents a summary of the ML based approaches with their important parameters, advantages, and limitations.

**Summary:**Table 2 shows that some ML-based approaches select the optimum subset of features to improve the detection accuracy, classify the network traffic into normal and abnormal behavior, or use ML algorithms for both purposes. However, several works did not provide details about feature selection technique used in their approach [3,8,10,21,45,50,51,53,54,56,60]. In addition, other studies, such as [7,8,21,45,50,51,53,59,60,62], did not mention the number of features used or selected in their approach. Moreover, two studies [57,60] did not disclose the type of program used in the implementation step, and did not provide information about the dataset and the feature selection technique used.

### 7.3. RQ3: What Are the Prevailing DL Approaches Contributed by Existing Studies to Detect RPL-Based 6LoWPAN Attacks?

This section presents a comprehensive review of the studies based on the DL mechanisms, as described in Section 7.1. In addition, we highlighted the essential findings from each study, followed by a summarization table that underlines the essential parameters while addressing the shortcomings of previous works. Then, we briefly review some of the neglected aspects of contemporary DL approaches.

Yavuz et al. [58] developed a DL approach for detecting routing attacks in IoTs. The proposed methodology has three stages: dataset simulation, feature preprocessing, and DL stage. The work used a self-generated dataset known as IRAD, using the Cooja simulator, and comprising three types of RPL routing attacks: HF, VN, and DRA. The authors implement different scenarios for each type of attack during the first stage. For example, the authors used various regular and malicious nodes in an HF attack and replicated the same process for other attack scenarios. Then, in the second stage, the authors employed different tools to aggregate and handle the collected information. The authors also performed some preprocessing on the extracted features, using the window size to calculate the values of the extracted features. Eventually, there were 18 extracted features, including packet features, control messages features, and new lists of features extracted from some mathematical calculation based on a specified time frame. The authors then used three feature selection methods, DT, Pearson coefficient, and histogram, to select important features. Next, the selected features are fed into the final stage for attack detection using a DL algorithm. A Multi-Layer Perceptron (MLP) classifier identifies normal and malicious behavior using different activation functions. For building the DL model, the authors utilized the sequential model and employed Mean Square Error (MSE) for the loss function. Furthermore, the authors selected the AdaDelta algorithm as the optimizer. The experimental results show that the AC of the DL model is 99.5% through the use of the Sigmoid function in the output layer. As for the proposed approach’s overall performance, the F1-scores for the model are 94.7%, 99%, and 95% in detecting DRA, HF, and VN attacks, respectively. Meanwhile, as for the AUC result, the model attained 94.2%, 98.1%, and 94.7% in detecting DRA, HF, and VN attacks, respectively.

An anomaly-based intrusion detection model using the DL approach for detecting malicious traffic in IoT networks was designed by [63]. The proposed approach sorts the network traffic into sessions and inspects the characteristics of network activities to identify five types of RPL attacks: BH, Opportunistic Service, DDoS, SH, and WH attacks. To achieve that, the authors employed three phases in the detection process: a network connection, anomaly detection, and mitigation. The first phase initialized and deployed the required network channel to sniff the network traffic. Next, in the second phase, the authors applied some processes, such as features extraction and transformation, to the data packets before injecting them into the ML module. After that, the ML module trained the data by employing a perceptual learning model known as a supervised ML algorithm.

Finally, the mitigation phase mitigates the identified attack and provides an adequate response by utilizing the actuator and handler module. Furthermore, this work uses the pre-trained layered Deep Belief Network (DBN) to develop the feed-forward Deep Neural Network (DNN) model, which is considered faster than supervised learning. Moreover, regarding the experimental result, the authors reported that the proposed approach obtained a significant result of detecting all types of attacks, for instance, in a DDoS attacks scenario, the proposed approach attained a Precision (96%) and Recall (98.7%) rate comparable to the result of the work [64]. Furthermore, the proposed scheme attained a higher F1-score of 0.973. Additionally, the authors implemented their approach on the IoT testbed, and the results revealed the Precision value (98.47%) and Recall value (97%) using actual sensors.

Kamel and Elhamayed [1] proposed a mitigation approach for minimizing the effect of IoT routing attacks on PRC in the healthcare environment. The proposed work uses a Convolution Neural Network (CNN) to discover five routing attacks: HF, SF, SH, WH, and VN. The architecture of the proposed approach comprises three layers, the medical data collection layer, the routing and network layer, and the medical application layer. In addition, the authors utilized a real-time dataset generated using the Cooja simulator. Finally, the Synthetic Minority Over-sampling TEchnique (SMOTE) was applied to deal with unbalanced samples in the generated dataset.

The authors employed three feature selection methods during the preprocessing step: weight by One Rule (One-R), chi-square, and weighted random Forest. Such methods are also used to solve other issues, such as noise and over-fitting. Then, they implemented the CNN model to identify suspicious behavior in network traffic. As for the experimental result, the results show that the proposed approach attained 96.87%, 94.85%, 3.13%, 99.65%, 93.8%, 97.19%, and 0.325 for AC, Precision, Error Rate, Recall, Correlation, F-measure, and Logistic Loss, respectively, for HF attacks. Overall, the proposed approach shows significant outcomes in terms of AC, Precision, Correlation, Recall, Error Rate, and Logistic Loss, leading to a reduction in PRC.

A DL approach for detecting Rank attack in RPL was introduced by [62]. The proposed approach employs MLP for verifying and classifying the normal and suspicious behavior of network traffic. In their work, the authors self-generated their dataset using the Cooja simulator. Moreover, during the implementation of the RPL attack in the data collection stage, the authors modified a set of parameters in the simulator’s configuration, such as icmpv6 and dag rpl files. Such modifications are necessary to initialize the RPL attack. In addition, the authors applied some preprocessing techniques, such as feature extraction, binarization, normalization, and sampling, to obtain a readable data format that are useable for training the MLP algorithm and testing the produced model.

Furthermore, throughout the creation of the MLP model, the authors used various inputs and outputs from the dataset. Moreover, various functions and parameters were used during the MLP implementations stage, such as ReLU being assigned as an activation function. To evaluate the proposed approach, the authors split the dataset into 80% and 20% for the training and testing phases, respectively. Regarding the experimental result, the proposed approach obtained 100% TPR and 24% FPR. Additionally, the model achieved 98%, 88%, and 92% for Precision, Recall, and F1-score, respectively, on the outcome of macro-Avg and weighted-Avg. Similarly, in terms of AC, the proposed approach attained a high performance of up to 96%.

Sahay et al. [65] developed a holistic framework to predict routing attacks in IoT-LLNs. They used several DL tools, such as the Graph Convolution Neural Network (GCNN) and Long Short-Term Memory (LSTM) model, to capture the spatial and temporal features of the IoT-LNNs. Furthermore, the authors used the smart contract-fortified blockchain technique to preserve the collected data from network nodes. Meanwhile, in the event of any suspicious behavior identified, such as the change of node’s rank exceeding the assigned threshold, the VN created by a non-sink node, and the number of DIS messages exceeding the pre-defined threshold, the smart contract generates a warning impulse. Another role of the smart contract is to obtain the required feature extraction and initialize the dataset and data visualization tool. Finally, the FNN model utilized the output from LSTM, GCN, and warning impulse from the blockchain-based smart contract for attack predictions.

The experimental results showed that the proposed framework achieved an AC of 94.50%, 86.13%, 82.46%, and 91.88% in predicting the normal scenarios, topological attacks, resource attacks, and traffic attacks, respectively. In addition, the authors claimed that their framework could detect and identify various routing attacks.

Molina et al. [5] introduced a DNN approach for detecting CID attacks in RPL networks. They used the Cooja simulator to implement CID attacks and three types of topological structures with a different number of normal and malicious nodes to build a dataset. The workflow of the proposed approach has three stages: data preprocessing, unsupervised pre-training, and supervised classification. The first stage comprises dataset balancing, value transformation, and scaling steps.

Regarding the implementation flow of the proposed approach, Firstly, the authors applied a Random Over-Sampling (ROS) technique to handle the dataset balancing, then implemented the One-Hot Encoding technique through the value transformation step to convert the feature format into a proper shape. After that, the standard scaling method was used during the scaling step. Later on, the authors implemented an autoencoder-based unsupervised algorithm to pre-train the second-stage model. Then, the DNN was used in the last stage to classify and identify normal and suspicious behaviors. Table 3 shows the summary of the existing DL approaches with their critical parameters, features, and advantages and limitations.

**Summary:**Table 3 clearly shows that most researchers employed synthetic datasets to evaluate their approaches and used Contiki (Cooja simulator) to simulate their experiments. Furthermore, it could be observed that there is a lack in the information provided in the reviewed studies, such as [65], where the authors did not disclose the feature selection techniques used in their works. In addition, [62] did not clarify the number of features used in their solution. Meanwhile, [58] generated a new dataset and publicly published it for the other researchers. Finally, [63] did not disclose the used dataset in their work.

### 7.4. RQ4: What State-of-the-Arts Combined ML and DL Approaches Have Been Used to Detect Attacks in RPL-Based 6LoWPAN?

This section thoroughly discusses the literature based on the combined ML and DL studies, as described in Section 7.1. Additionally, we highlight the essential findings from each study. After that, we provide a summary table that highlights the critical points while resolving the inadequacies of prior works. Finally, we provide a brief overview of some neglected characteristics of the presented combined approaches.

Cakir et al. [30] proposed a DL approach for detecting and preventing HF attacks in RPL. In this work, the authors implemented a Gated Recurrent Unit (GRU) network with Recurrent Neural Network (RNN) to identify legitimate and malicious nodes. The methodology of the proposed model comprises three steps: network simulation, data preprocessing, and attack detection. In the network simulation step, the authors carried out three scenarios with different legitimate and malicious node combinations. The authors also computed each node’s PRC parameters for later use as features to detect the attacks in the detection step.

For preventing HF attacks, the authors designed a set of rules that depends on the Rx power value of the network nodes. In particular, the maximum and minimum Rx values of legitimate node(s) are used to identify malicious nodes. As a result, the proposed model achieved the highest AC values of 99.96% and 99.90% in the third scenario, using five and four features set, respectively. Furthermore, the authors attained the best outcomes (lowest value is best) for ACC, MSE, and MAC at 0.01, 0.05, and 0.05, respectively, in the third scenario with four sets of dataset features.

In [66], the authors presented an ML approach for detecting attacks in RPL-based IoTs. The proposed approach is designed to detect a combination of various RPL attacks, such as Rank and VN attack, Rank and Sybil attack, Rank and BH attack, and Decreased Path Metric attack. The authors evaluated the vulnerability of the most popular RPL’s Objective functions (MRHOF and OF0) against invoked attacks in their work. In addition, the authors performed data preprocessing, feature selection and reduction, sampling technique, and normalization, which are essential to getting the best attack detection result. Moreover, the authors initialized their proposed dataset based on IoTs features and network metrics to test the performance of the proposed approach. The generated dataset comprises 24 features collected based on network and power metrics. Furthermore, the authors used several classification algorithms, such as NB, SVMs, MLP, RF, and ZeroR classifiers.

Regarding the simulation scenarios, the authors ran seven experiments. Some experiments were performed using a complete set of features, while the rest used an optimal set of features.

As for the results, the SMOTE-MLP and Subsample-NB obtained the best results compared to other classifiers in the first experiment. Moreover, from the second to the fifth experiment, the SMOTE-MLP reported superior results compared to the others. Additionally, in the sixth experiment, the ensemble model (Voting-MLP and RF) acquired the top results in RSME, Mean Absolute Percentage Error (MAPE), ROC average, and correctly classified instances compared to the other models. Moreover, the seventh and eighth experiments, the MLP network and MLP network metrics produced the best results when using the OF0 type. Finally, the overall performance of the ML algorithms shows that the voting (MLP and RF) classifiers gained excellent outcomes compared to other ML algorithms.

The authors in [67] introduced ML and DL techniques to identify an optimal subset of features for identifying routing attacks in RPL based IoT networks. The architecture of the proposed approach encompasses several stages. First, the authors generated an IoT routing dataset using the Cooja IoT simulator. Then, in the second stage, the preprocessing technique was applied to prepare the collected data; additionally, in this stage, the SMOTE was employed to solve the balancing issues of abnormal samples. Furthermore, the authors divided the dataset into four blocks randomly. In addition, for the feature selection stage, the Cuckoo Search (CS) meta-heuristic algorithm is employed to select the optimal features. Moreover, in this work, the Dagging meta classifier is applied to enhance the performance rate of the base learner Bayesian Logistic Regression (BLR) by minimizing the over-fitting problem. Additionally, the modified version of the CS algorithm was carried out using BLR-based Dagging, which is better than the original CS algorithm in terms of speed, convergence rate, and iteration number. Lastly, the authors utilized SVM, CNN, and Fuzzy Unordered Rule Induction Algorithm (FURIA) in the classification stage.

Regarding the implementation of the presented work, the authors conducted two experiments. The first experiment examined the impact of Dagging on the original CS algorithm and BLR, and the second experiment inspected the performance of the standard CS algorithm with BLR only. Meanwhile, after implementing the designed scenarios, the authors obtained 12 features from the first experiment and 15 from the second. Additionally, the outcomes revealed that employing BLR-based Dagging improves the original CS algorithm’s speed and enhances the performance of BLR.

As for the result, the first experiment showed that the CNN model attained the best results of 98.57%, 1.43%, and 98.32% for AC, Error, and F-measure, respectively. Moreover, the results of the second experiment exhibited that the CNN also obtained the highest values, 92.57%, 7.43%, and 90.4% for AC, Error, and F-measure, respectively. The results showed that the first experiments’ outcomes were better in AC, Error, and F-measure than the second.

Bokka and Sadasivam [32] proposed ML techniques for detecting routing attacks in RPL-based IoT networks. The authors utilized a synthetic dataset generated using the NetSim simulator in their work. The collected network’s traffic includes normal and malicious behaviors. In addition, the authors implemented seven types of RPL attacks, SH, DIO Suppression, BH, SF, Sybil, and DIS Flooding attacks. Moreover, the gathered datasets contain 21 attributes and two labels reserved for normal and attack classes. The architecture of this work comprises several modules, such as data collection, feature selection, data preprocessing, data splitting, and ML classification algorithms.

The implementation flow of the proposed approach starts by collecting network traffic and then employing the feature extraction step. After that, the authors employ a random forest algorithm to reduce the datasets’ features and select the most powerful ones. Later, the preprocessing techniques, such as cleaning, encoding, and scaling, are applied to the selected features. Finally, the selected features are evaluated using seven kinds of ML classifiers, such as MLP, KNN, AdaBoost (AdB), RF, Gaussian Naive Bayes (GNB), DT, and LR.

As for the experimental results, the authors reported that the DT classifier obtained the best AC, Precision, and F1-score, i.e., 92.6%, 0.946, and 0.955, respectively, for 10% test data hold-out. On the other hand, the LR, GNB, and MLP achieve the best result of 1.00 for all cases regarding the Recall value. Moreover, the RF classifier gains the best AUC of 0.946 in the case of 20% hold-out data. In contrast, the GNB classifier attains a low AUC value of 0.623 during the 10% hold-out dataset among other classifiers.

Medjek et al. [35] developed a fault-tolerant AI-based IDS for detecting routing attacks in RPL-based IoT networks. The authors targeted six different types of RPL attacks: DRA, BH, SH, HF, SF, and VN attacks. In addition, the authors used the Cooja simulator to generate two types of datasets, two-class (normal with attack) and multi-class (normal with six attacks). Moreover, the authors obtained the configuration settings used in [58] to set up their simulation environment for the data collection process. The architecture of the proposed approach consists of several modules, such as data collection, feature engineering, selection, and classification.

As for the feature selection process, the authors combined two types of embedded methods: RF and a filter method, Pearson Correlation (PC). Then, they selected the top 10 most essential features produced by RF. On the other hand, the authors applied the correlation matrix using the PC method, and the authors defined a threshold value of 0.3 for the correlation. Consequently, the features that achieve the defined value will be selected. Afterward, the authors employed an intersection step to produce a new subset of features. Concerning the classification stage, the authors used six types of ML classifiers: DT, RF, KNN, NB, MLP, and LR. Moreover, the authors also used the Sequential DL model to evaluate their approach.

As for the experimental result of the proposed approach, the KNN, RF, and DT classifiers performed the best for all metrics compared to the other classifiers. Furthermore, the DT, RF, and KNN gained more than 99% classification AC for both class types (binary and multi-class). Moreover, in multi-class classification, the results reveal that KNN obtains an AC and DR of 99% and 98%, respectively. Furthermore, the RF and DT gained 98% of AC and DR. Consequently, based on the attained result, the authors conclude that the RF model is best in performance and fitting time, making it the most suited for intrusion detection in RPL-based networks. Finally, the authors produced an RF-based IDS approach, introducing fault tolerance and intrusion tolerance for detecting attacks in RPL-based Industry 4.0 networks using an RF classifier.

Lastly, a study by [68] introduced an ML approach based-IDS for detecting RPL attacks. The architecture of the proposed work is based on hybrid IDS, which combines anomaly-based and signature-based IDS. In addition, the 6LoWPAN compression header is used in this work to study and observe the form used during routing attacks. Furthermore, the obtained rule or signature from the ML algorithm will be injected into the border router. Moreover, this work implemented different types of RPL routing attacks, such as HF, WH, and SH attacks. The methodology of the proposed approach comprises three stages: 6LoWPAN network traffic, data preprocessing, and data classification. First, the authors generate a dataset using the simulation environment for different scenarios of RPL routing attacks. Then, the authors applied some preprocessing and analysis on the collected data to extract the features from network traffic.

Such features include 24 features representing regular and attack node traffic characteristics. After that, the authors applied some techniques to extract the relevant features that contribute to the detection of RPL attacks. Moreover, the authors use a Particle Swarm Optimization (PSO) algorithm, a type of bio-inspired optimization algorithm. Such an algorithm is used to optimize the optimum parameter of classification algorithms that are used in stage 3. The optimization step plays a vital role in obtaining the best performance of ML classifiers, which enhances their efficiency in differentiating between normal and suspicious samples of the proposed dataset features.

As for stage 3, the authors use several classification algorithms, such as NB, SVM, RF, KNN, and a False MLP, to classify the normal and malicious behavior. The experimental results show that the RF attained the best result in detecting all kinds of attacks compared to the other algorithms. Table 4 summarizes the existing combined ML and DL approaches with their significant parameters along with advantages and limitations.

**Summary:** Based on our analysis of the studies presented in this section, we discovered that two studies, [30,66], did not provide details about the feature selection technique used. Furthermore, it is evident that all studies used synthetic datasets to test their experiments, except for one [67], which used the IoT Routing Dataset.

### 7.5. RQ5: What Are the Recent Applications Based on ML and DL Approaches Proposed for Detecting Attacks in RPL-Based 6LoWPAN?

This section presents the most recent applications based on ML and DL approaches proposed for detecting attacks in RPL networks.

Using ML methods, Zahra et al. [69] proposed a framework for detecting routing attacks in the RPL network—specifically Rank and WH attacks—using three modules: profiling, model building, and model testing. The first module investigates the factors and parameters of Rank and WH attacks and identifies the differences between those factors to detect each type of attack. Furthermore, the parameters of Rank and WH are assigned to be used later in building an effective and high-performance detection model. At the same time, the second module selects an appropriate set of ML algorithms to detect the attacks accurately. Then, various ML algorithms are evaluated using the predefined parameters of Rank and WH attacks, and the best ones are selected to be a part of the detection model. After that, the selected algorithm is used to build and train the detection model. The last module tests the performance of the proposed model in detecting Rank and WH attacks, then finally analyzes the results.

Another work by Zahra et al. [70] extended their previous work [69] by introducing a mitigating approach. The authors used a similar methodology to their previous work to design the attack detection model. In addition, the obtained results from the detection model were stored in an index for further analysis and used to initialize the mitigation scheme. Unfortunately, the authors failed to provide more detailed information about the mitigation scheme. Finally, the authors proposed SVM, an ML model for attack prediction.

An ML approach based on the SVM classifier to detect DoS attacks in IoT was proposed in [71]. The proposed architecture designed a real-time data collection tool to monitor the network behavior and collect IoT network datasets. The workflow of the proposed architecture contains three models: Data Collection Model (DCM), Detection Model (DM), and Classification Model (CM). The DCM is designed to work with any IoT protocol type and collects IoT communication data from three layers—physical, network, and application layer features.

The physical layer contains features related to detecting jamming attacks that target this layer. Next, the features at the network layer are crucial for detecting many prevalent attacks, for instance, BH and DRA attacks. Finally, the application layer’s features are related to some applications, such as humidity, temperature, and node power level.

The detection process of the attacks begins with DCM. Then, the generated dataset from the DCM will be utilized for training and testing the proposed ML algorithm. Furthermore, the DCM includes a two-stage scenario. The first stage constructs the ML model according to the collected dataset, and the second stage embeds the predicted model. As for the deployment of the proposed approach, the authors implemented a scenario in the Cooja simulator, where the system comprises two types of IDS nodes: IDS agent and backbone (6BR router) IDS. First, they deployed the IDS agent to sniff and monitor network traffic. At the same time, they installed other IDS nodes in the 6BR router to make the final decision and send orders to the network nodes if any suspicious behavior is detected.

Al-Hadhrami and Hussain [72] introduced a framework to build a real-time dataset comprising three known RPL attacks: Flooding, SH, and BH attacks. The authors proposed a queuing method for collecting the network traffic using several sniffing nodes deployed in the network. In addition, the authors extracted the features from three different layers: physical, network, and application layers.

The framework comprises four scenarios with the same topology. The first scenario represents the normal network behavior without any attack. In contrast, the other three scenarios contain normal and attack behavior. The dataset generation procedure comprises seven modules and units. The first unit focused on initializing the network and setting up the attack scenarios. Throughout this module, the authors utilized industrial IoT sensors that have constrained resources and communicate using IEEE802.15.4 and 6LoWPAN protocols.

Next, the traffic generation module used the simulation program, Cooja, to generate the traffic. The sensor nodes are programmed to run humidity and temperature applications, enabling the capturing algorithm to collect application-layer features. Capturing data from the network requires the authors to use props packets that are distributed equally in the network. Such packets continuously monitor the network and send the gathered data to the aggregation module. The data aggregation module accumulates the collected data and checks for duplication in the information by inspecting each packet’s node ID and timestamp. The aggregated data are forwarded to the queue system to check the time window. The packets located in the same time window are sent to the feature extraction unit. The feature extraction unit is also designed to take the packets from the queueing system and extract the features. The authors extracted the features from three different IoT layers in this work. The first vector of features includes physical layer features, such as signal strength and transmission range. In comparison, the second feature’s vector involves the network layer features, most of which are linked to network attacks. The last feature’s vector contains application layer features related to humidity, temperature readings, and node power level.

Another work proposed by Al-Hadhrami et al. [73] focused on building a Data Exportation Framework (DEF) for IoT devices. The proposed approach aims to extend the existing features of the Cooja simulator program by adding an extension to the current Collect View plugin. The proposed DEF tool enables the simulation program to export the data into different formats, such as CSV files or MySQL databases. The authors reverse-engineered and modified the existing code of the Collect View plugin model to run the DEF tool during the runtime.

The DEF framework comprises four modules: Data Handler Module (DHM), Data Visualization Module (DVM), Database Management Module (DMM), and API Module (APIM). The DHM is responsible for extracting collected data from each node and archiving it as an ArrayList for presentation later in the table. The DVM operates as a database interface for reading and writing into a database. Next, the APIM provides developers with a simple API that includes database connection and data retrieval.

As for the evaluation of the DEF framework, the authors implemented six scenarios, three with the DEF tool and three without it. The result illustrated a slight increase in CPU and memory utilization during the implementation of DEF scenarios, which caused a little overhead on the resources.

Finally, Essop et al. [74] provided a dataset generation approach for anomaly-based IDS in IoT and IIoT networks, using the Cooja simulator to generate comprehensive IoT/IIoT datasets. The generated dataset from the simulated scenario captures information from Contiki plugins, such as "powertrace", which contains features related to the energy consumption of IIoT devices, such as CPU consumption and Low Power Mode (LPM). The Contiki tool, known as "Radio messages", contains network traffic features employed in the generation process of the IoT/IIoT dataset. In addition, the authors generated four datasets in this work; two of them contain benign traffic, and the other two comprise malicious traffic. In both benign and malicious scenarios, the authors generated the datasets from "powertrace" and "Radio messages" separately. Moreover, in the malicious scenario, the authors implemented UDP Flooding attacks. Furthermore, during the generation process, the authors computed the total energy consumption of the nodes.

### 7.6. RQ6: What Are the Existing Threats in RPL-Based 6LoWPAN That the Existing Studies Had Addressed?

To answer this question, we first classify the attacks using the same taxonomy as shown in Figure 1; we provide a brief description of each type of attack. Then, we present a visualization of the attack’s statistics in the existing studies, as discussed in Section 7.2, Section 7.3 and Section 7.4. The following subsections illustrate the attacks according to their target.

#### 7.6.1. Resource-Based Attacks

- **Hello Flooding (HF) Attack:** This attack aims to make the network services or resources unavailable. The HF attack is carried out by constantly flooding the network with many "Hello" packets to notify their one-hop neighbors of their presence. The high transmission power of the malicious nodes will persuade all other nodes in the network that it is their neighbor [49,54]. Meanwhile, the adjacent nodes will respond to those messages. As a result, massive network traffic will be generated, resulting in control overhead, service unavailability, instability, and node resource depletion [56].
- **Increased Rank (IR) Attack:** In the IR Attack scenario, a malicious node increases its rank illegitimacy. Meanwhile, the malicious node announces itself near the root node, but with a higher rank and worse path. Therefore, nodes in the malicious node’s subtree and those in its proximity must choose other nodes as parents, leading to more delay and disruption of the routing topology [14,65].
- **Rank Attack:** In a Rank attack, the attacker distributes the minimum rank r-value, intending to become a parent node. In the RPL network, the parent node is chosen based on the rank metric. Regularly, the nodes closest to the root node have the lowest rank. Therefore, using a low-rank value, the rank attacker is chosen as a parent, and the other nodes forwarded routing messages along the network’s attacking path. Thus, it loads extra overhead and excessive energy dissipation at the nodes, resulting in lower routing performance [59,75].
- **Version Number (VN) Attack:** In a VN attack scenario, the attackers target the global repair feature of RPL by modifying the version number of the existing DODAG. The root node is responsible for changing the version number in normal operation. However, suppose the malicious node transmits a DIO message with a higher version number. In that case, it forces the global repair mechanism to start and reconstruct the DODAG, which will result in additional overhead and drain the nodes’ power resources [14,51].
- **Local Repair Attack:** The rogue node increases its rank to infinity during the execution process. It transmits this message to the entire network, compelling other legitimate nodes to look for a new parent to reach the root (gateway) node. When this occurs frequently, network performance suffers, as the topology must be modified every time the node changes [63].
- **Increased Version (IV) Attack:** In this attack, the fraudulent nodes purposefully change the version number of the DIO control packet and send the altered DIO message to their neighbors. When the neighbors receive the altered DIO message, they demonstrate their exclusion from the new DODAG, resulting in the unnecessary reconstruction of already-available DODAG. Consequently, the frequent reconstruction increases network traffic and causes an impact on critical network factors, such as lifetime, availability, and energy efficiency [56,76].
- **DIS Flooding Attack:** This attack occurs when one or more malicious nodes periodically send DIS messages to neighboring nodes within their transmission range, and upon receiving of DIS message, the trickle timers of the victim node(s) would reset, and this process continues until the power resources of the victim node(s) depleted, crashing the network [62].
- **DDoS Flooding Attack:** In a DDoS flooding attack, various malicious nodes target the network nodes with vast amounts of traffic to interrupt the normal operation of the network services. This attack also increases communication overhead and overwhelms the power resources of the sensor nodes [63,76,77].

#### 7.6.2. Topology-Based Attacks

- **Selective Forwarding (SF) Attack:** This attack happens when the malicious node dislocates the network routing path by selectively forwarding some of the packets in the network while leaving the rest forwarded to the original destination [21]. In addition, this attack can involve one or more malicious nodes and could either be consecutive or non-consecutive [23].
- **DIO Suppression Attack:** The purpose of the DIO suppression attack is to disrupt or slow down the network’s transmission of DIO messages. For this purpose, Trickle’s DIO suppression method is used. During this attack, the adversary continuously sends a DIO message that the receiving nodes regard as consistent. Suppose the nodes get a sufficient number of consistent DIOs. In that case, they disable their own DIO transmission, resulting in a general decrease in the quality of the routes or, in the worst-case scenario, a network breakdown [62,78].
- **Worst Parent (WP) Attack:** In the WP attack scenario, the attacker fabricates routing information and broadcasts DIO messages to neighboring nodes with different rank values than genuine ones. Later, the child node assigns the malicious node (with the highest rank value) as their parent instead of the best ones specified in the usual RPL scenario. As a result of this attack, the network nodes suffer from non-optimal routing paths, degrading their performance and leading to high consumption of power resources [3,24].
- **Opportunistic Service Attack:** In this attack, the malicious node gains its trust value by initially offering highly dependable services and then later resorts to providing inferior services for its own sake [63].
- **Temperature Level Attack:** The attacker manipulates the reading of the temperature and humidity level sensors in patients’ rooms. Consequently, the air-conditioning system stabilizes the temperature level based on erroneous data, which could cause deterioration of the patient’s health [7].
- **Heart Attack:** This is an e-health related data attack that manipulates the patient’s heartbeat level information. Such modifications might cause bad decisions being made or ignored by emergency personnel, such as when a patient’s heart rate is extremely low/high and quick medical attention is required [7].
- **WormHole (WH) Attack:** In a WH attack, two or more attackers collaborated to establish a virtual tunnel between them to pass the traffic, entirely or selectively, through it instead of its original route. Therefore, such an attack disrupts the network topology, exhausts network resources, and provides the attackers with access to sensitive information [69,79].
- **SinkHole (SH) Attack:** A malicious node broadcasts itself as the best convenient route (optimal path) to be a preferred parent for the surrounding nodes. Then, the network traffic of the child nodes will be forwarded to the SH node. Therefore, this attack disrupts the communication and leads to other kinds of attacks [32,49,80].
- **BlackHole (BH) Attack:** In a BH attack, the pernicious node announces itself as the shortest route to the destination. All the packets arriving at this node will be dropped and, thus, prevented from reaching their destinations. Therefore, this attack will create a ‘hole’ in the network without the senders being aware of their packets’ delivery status [49,63].

#### 7.6.3. Traffic-Based Attacks

- **Decreased Rank (DRA) Attack:** In this attack, a rogue node broadcasts its fabricated rank to its neighbors, resulting in neighboring nodes choosing the fraudulent node as their parent. Consequently, this causes other nodes to route their messages through a fake node. In addition, the fraudulent node broadcasts its predecessor’s rank as its own to deceive other nodes. The main effect of this attack is to increase the network’s traffic, and it can also be used to eavesdrop on DODAG’s downward nodes [56,81].
- **Sybil Attack:** In the Sybil attack scenario, the attacker masquerades the identities of multiple legitimate nodes to access network data. This attack deteriorates the network’s performance and increases the control communication overhead, resulting in delegated power resources. In addition, this attack could serve as a jumping-off point for other types of RPL attacks [49,82].
- **Clone ID (CID) Attack:** In this attack, the attacker takes the ID of one existing legitimate node and transfers it to the malicious node, resulting in the data being routed to the malicious node instead of legitimate nodes. Therefore, the attacker will sniff a large size of the network information [49,83].

**Figure 8:**
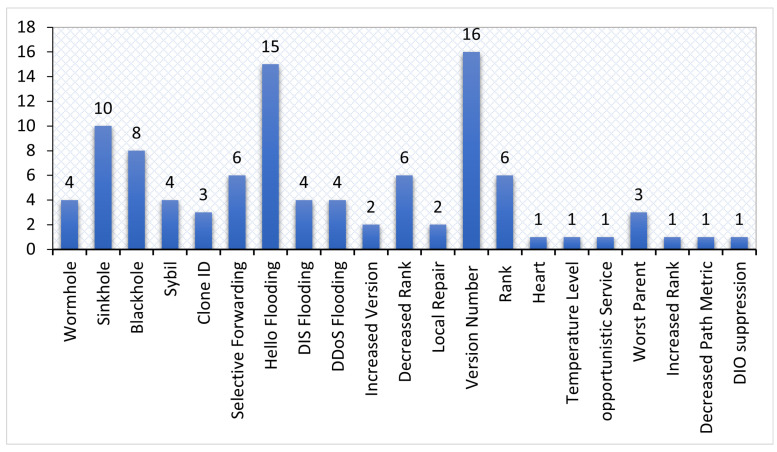
Distribution of the attacks in the existing studies.

Figure 8 shows the distribution of attacks in the reviewed studies, as discussed in Section 7.2, Section 7.3 amd Section 7.4. It is evident from Figure 8 that the VN and HF attacks are the most frequently studied, followed by SH and BH attacks. In addition, the DR, Rank, and SF attacks can be found in six studies. However, the rest of the attacks received very little attention from researchers. Therefore, it could be concluded that those attacks received less attention because they are fairly easy to detect or too challenging to implement in RPL networks. To sum up, the analysis of implemented attacks in the existing studies would help future researchers detect such attacks and focus their attention to new kinds of attacks.

### 7.7. RQ7: What Tools and Network Simulators Are Used in the Existing Studies, and What Are the Occupied Evaluation Metrics and Parameters in the Reviewed Studies?

**Figure 9:**
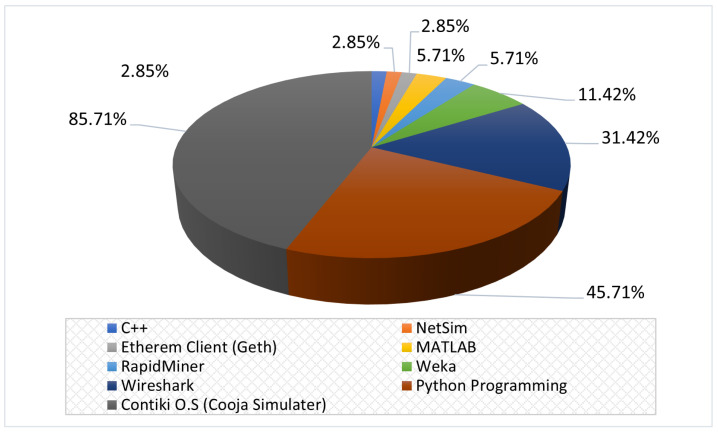
Distribution percentages of the used tools and network simulators in the existing studies.

**Figure 10:**
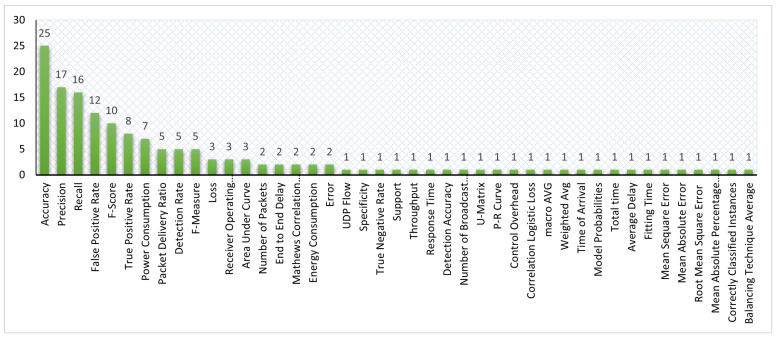
Distribution of the used metrics and parameters in the existing studies.

To answer this research question, we extracted the available information from the reviewed studies in Section 7.2, Section 7.3 and Section 7.4. We then present the analysis of the tools used and the evaluation metrics utilized in Figure 9 and Figure 10, respectively.

As for the used tools, it is evident from Figure 9 that nine different tools and network simulators are used in the reviewed studies. However, two studies did not disclose the tools used in their work. Most studies used the Contiki O.S (Cooja simulator) (85.71%), followed by Python programming (45.71%), and Wireshark (31.42%). Meanwhile, 11.42% of the studies used Weka tools in their work. It seems that researchers working in this field rarely utilized other tools and platforms in their studies.

Our analysis shows that the Cooja simulator is the most popular program among researchers for simulating network traffic (normal and malicious traffic) and data collection processes. Meanwhile, Python programming is the programming language of choice for most researchers for data preparation, feature selection, and classification stages. Moreover, the role of the Wireshark program in the reviewed studies is to collect the information from the simulation environment and export the collected data into readable forms.

From Figure 10, it is evident that the most used metric is Accuracy, which was found in 25 studies, followed by Precision and Recall, used in 17 and 16 studies, respectively. The F-score and False Positive Rate also obtain significant consideration in 12 and 10 studies, respectively. However, few researchers evaluated their approaches using rarely known metrics such as Fitting Time, Balancing Technique Average, and MSE. On the other hand, some researchers evaluated the performance of their mechanism using well-known data mining metrics, such as AC, Precision and Recall, extracted from confusion metrics’ parameters. Moreover, other researchers are also evaluating and validating the proposed approach for detecting the attacks using a set of network parameters to measure the effectiveness of the proposed defense mechanisms under RPL attacks, such as PDR, E2E Delay, and PRC.

### 7.8. RQ8: What Are the Datasets Utilized to Evaluate the Existing Studies, and Are There Any Available Datasets Designed Specifically for RPL-Based 6LoWPAN?

**Figure 11:**
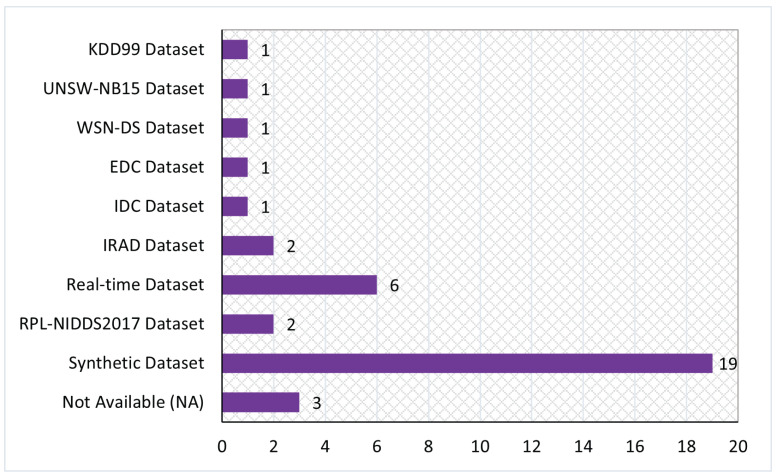
Occurrence of the datasets in the existing studies.

Responding to this question, we extracted the dataset types used based on information provided in the reviewed studies presented in Section 7.2, Section 7.3 and Section 7.4. The result is shown in Figure 11, in which 19 studies used synthetic datasets, 6 used real-time datasets, and 3 studies did not state the dataset type used in their experiments. In addition, two studies used the IRAD and RPL-NIDDS2014 datasets. Finally, other types of datasets, such as IDC, EDC, WSN-DS, UNSW-NB15, and KDD99, were only used in one study, respectively.

It is apparent from Figure 11 above that most researchers use synthetic datasets to evaluate their mechanisms. However, such datasets are typically generated to cater to specific tasks or configurations that might not apply to real-world environments. Furthermore, due to privacy and security concerns, most researchers prefer to generate and use their own private datasets to evaluate their works. However, since there is no standard configuration for setting up the network and simulation scenarios, it is challenging for researchers to compare their approach with others fairly. The following list provides more details about the existing datasets that are made explicitly for RPL-based 6LoWPAN IoT networks:

- **RPL-NIDDS2017 Dataset** is a synthetic dataset created by Ranga and Verma [46] in 2018 using the NetSim program to simulate various network scenarios. They simulated an IoT network scenario comprising sensor nodes, a gateway, routers, and wired nodes to generate the dataset, containing 20 attributes and two additional attributes for labeling. In addition, the dataset included seven attack traces: Local Repair attacks, CID, BH, SF, Sybil, HF, and SH attacks. Moreover, the dataset’s features were divided into three types: flow, basic, and time. They also proposed an approach to detect the attacks, as mentioned in Section 7.2.
- **IoT DDoS Dataset:** Yahya Al-Hadhrami and Hussain [72] proposed a real-time dataset explicitly designed for the 6LoWPAN/RPL network. The dataset contained three DoS-based RPL attacks: DIS flooding, SF, and BH attacks. Twelve features were extracted from the physical, network, and application layers. In addition, the authors devised queueing methods for collecting network traffic from a set of sniffing nodes. The dataset simulation was executed for 24 h, resulting in more than 4,195,537 packets of RPL-based 6LowPAN network traffic. The simulation process involved 29 nodes, where the Zolertia (z1) nodes are mimicked in the Cooja environment. Moreover, to reflect the environment of real-world networks, the authors included two distributor nodes that generate a noisy signal at predetermined intervals. Furthermore, the proposed system that generates the IoT DDoS dataset consists of four components: the capturing medium, data aggregation, queuing unit, and the feature extraction unit. The authors conducted four scenarios. The first scenario represents the normal network behavior without any attack, while the rest represent the three DoS-based RPL attacks.
- **IDC and EDC Dataset:** The researchers [7] at the SERCOM Lab of the University of Carthage have created two datasets, IDC and EDC, reflecting a smart hospital infrastructure. The generated IDC dataset comprises the normal and malicious behavior of network traffic. The malicious behavior includes the traces of three attacks: Rank, Flooding, and VN Modification attacks. Meanwhile, the dataset is split into training and testing sets, with 1000 instances utilized for the training set and 200 for the testing set. The EDC dataset generation used two types of data, environmental and body sensor data, where the environmental data include environmental information, such as temperature, light, and humidity. The training and testing set of environmental data contained 100 and 200 instances, respectively. The body sensor data comprise body temperature information and heart rate information. Similar to the environmental data, 1000 instances were utilized as a training set, and 200 for the testing set.
- **IRAD Dataset:** Osman et al. [31] developed a VN attack-based dataset. The authors developed a Python model to extract the dataset features. The total number of extracted features was 113. They proposed a dataset comprising 1,050,861 records, out of which 884,861 were assigned as benign and the rest as malicious traffic. In addition, the authors developed a lightweight mechanism to identify the attack, as stated in Section 7.2.

## 8. Open Issues, Challenges, and Future Research Directions

**Figure 12:**
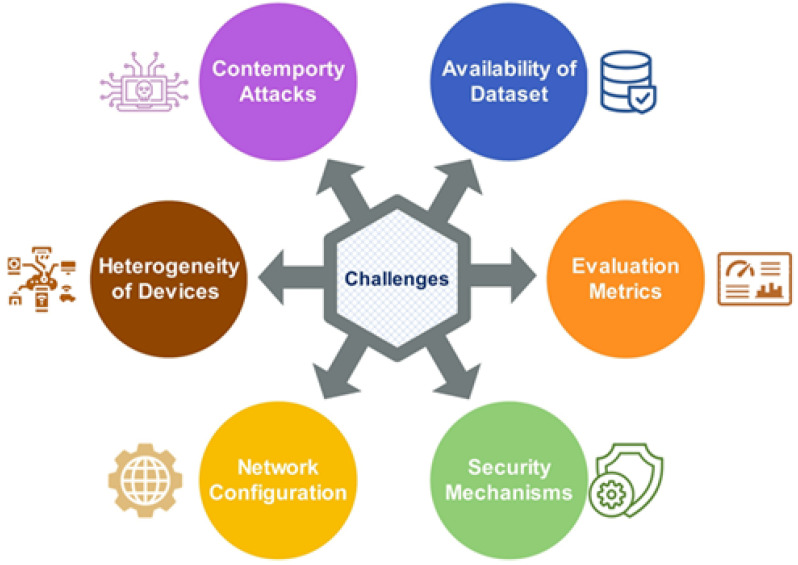
Issues and challenges.

This section presents the research issues and challenges faced by ML, DL, and combined ML and DL approaches to provide future research directions in developing new defense mechanisms for RPL attacks. Figure 12 illustrates the issues and challenges.

The following points provide more details about those challenges:

- **Challenge 1—Datasets Availability:** This study discovered that most researchers use self-generated datasets from various simulation programs, either synthetic or real-time, to evaluate their approaches [7,47,48]. However, some researchers do use existing publicly available datasets [31,49], even though many of those datasets were based on traditional networks’ traffic, which is different from the traffic of RPL-based 6LoWPAN networks [49]. Unfortunately, only a handful of researchers constructed datasets for RPL networks and made them available publicly [46,58,72]. Consequently, it is difficult for researchers to compare their work with others due to the variations in the datasets used.
- **Challenge 2—Evaluation Metrics:** We found that most studies used well-known evaluation metrics and parameters to validate the effectiveness of their work. Unfortunately, those parameters are commonly used in devising mechanisms for detecting attacks in traditional networks. In addition, due to the constrained environment of IoT networks, there is a need to also evaluate the proposed mechanism in terms of network and nodes metrics, such as PRC, PDR, and E2E delay. However, only a few researchers utilized these metrics in their studies [35,66,67]. Consequently, that might lead to bias in the results.
- **Challenge 3—Implementation of Security Mechanisms:** We can infer from our observation that the majority of researchers applied traditional security solutions to detect attacks in RPL-based 6LoWPAN networks [30,32,66]. Due to limited device capabilities in RPL-based 6LoWPAN networks (e.g., processing, memory, and power), there is a need for lightweight security and robust mechanisms with low complexity to avoid depletion of network resources and minimize the response time to defend against possible attacks.
- **Challenge 4—Network Configuration:** We noted many variations in the network configuration parameters used by researchers, such as the number of normal and malicious nodes, network topology, and network size. Without a standard configuration, researchers will face challenges when comparing their work to others. Furthermore, the evaluation scenarios used by the majority of existing approaches were using a small network [47,52,53]. However, in reality, IoT deployment is typically a vast network comprising various resource-limited nodes. Consequently, in such a network, the actual performance of the existing solutions may degrade and decline, making it less effective and vulnerable to attacks.
- **Challenge 5—Diversity of Devices:** This study discovered that the majority of approaches were designed and tested with only one or two types of sensor nodes (see Section 7.2, Section 7.3 and Section 7.4), i.e., the interoperability of non-homogeneous devices is one of the first assumptions of IoT applications. However, different hardware configurations can impact the performance of routing protocols and message processing rates. Therefore, researchers creating IoT routing solutions must consider the heterogeneity of hardware components.
- **Challenge 6—Contemporary Attacks:** We inferred those new attacks are technically and behaviorally different from the earlier ones. ML and DL models are usually trained with more outdated datasets’ features. However, new attacks might require a different set of features to identify. Consequently, the new attacks may either evade classifiers, generate false alarms, or reduce detection rates.

In addition, most studies superficially describe the pseudo-codes of the attacks, making reproducing them difficult. Furthermore, the persistent change in attack variations and the considerable increase in attack volume add to the challenge of identifying and responding to those incidents and threats. Moreover, several RPL-specific attacks are yet to be identified, thus requiring substantial defense mechanisms. Unfortunately, there are very few attempts to develop defense mechanisms against sophisticated attacks [14,16].

Based on the outcomes of this SLR and our standpoint, we provide the following future research directions for ML- and DL-based approaches in detecting RPL attacks:

- There is an urgent need for a standard comprehensive benchmark dataset that is publicly available to enable researchers to test the performance of their proposed works and compare them with others fairly. The proposed solution will address **Challenge 1**.
- Since RPL networks comprise low-powered nodes, there is a need to evaluate the performance and impact of the existing solution using additional network parameters, such as PRC, computational cost, deployment strategy, and coverage area of the defense mechanism. Furthermore, there is a need to develop lightweight ML and DL approaches that operate in a constrained environment and are adaptable for deployment in tiny devices. The proposed solutions will tackle **Challenge 2**.
- Researchers need to develop efficient mechanisms in dynamic network topology and support mobility options. In addition, the deployment of the detection mechanism in the network plays a crucial role in detecting the attacks successfully. Hence, there is a need to identify the optimum location in the network that contributes to a high detection rate with less energy and result in the lowest computational overhead for network nodes. The suggested solution will address **Challenge 3**.
- Researchers need to develop highly scalable and fast response solutions that provide a minimal delay in information transmission, especially for crucial IoT applications. This suggested solution will address **Challenge 4**.
- There is a lack of solutions that work with heterogeneous network devices. The expected future of IoT networks is towards technological convergence with different technologies, such as cloud computing, software-defined networking, blockchain, and 5G. The offered solution will solve **Challenge 5**.
- There is a need for multiple defense approaches for guarding against newly discovered RPL attacks. Furthermore, there is also a need to incubate combined/hybrid ML and DL approaches to exploit their powerful features to identify known and zero-day threats. The proposed approaches must be highly robust, scalable, and support Quality of Service (QoS). The offered solutions will address **Challenge 6**.

## 9. Conclusions and Limitations

This study conducted a comprehensive systematic literature review on existing ML, DL, and combined approaches proposed to detect attacks in RPL-based 6LoWPAN networks. The reviewed studies were from five databases published from January 2016 to mid-2021. After several filtering stages, the final count of articles selected for the review process was 49. Then, we analyzed the existing studies from the 49 articles to answer our defined research questions related to bibliographical info, techniques, programs, tools, performance evaluation metrics, datasets, and limitations. The findings reveal that the number of publications within the RPL domain has increased rapidly in recent years. Most researchers have published their work in the IEEE Xplore® database in the form of journaled articles. Afterward, we discovered that most studies focused on ML mechanism implementation with promising results, followed by DL and combined approaches. Furthermore, we noted that the VN and HF attacks are the most prevalent in the existing studies. In contrast, some attacks received less attention from researchers, which might threaten the RPL networks.

On the other hand, we realized that most researchers used Contiki OS (Cooja Simulator) to implement their proposed approaches. As for the metrics used, most researchers computed accuracy metrics in their work, which signifies its importance in the performance evaluation stage. However, we also noticed that most studies ignored some crucial parameters, such as models, approaches, and systems, in evaluating their proposed solutions. As for the datasets, many researchers did not disclose the dataset used in their works, but those that do mostly use synthetic datasets.

Additionally, some researchers use public datasets created using traditional network traffic, which is unsuitable for RPL-based 6LoWPAN. In this regard, we gave some insights into the used and available datasets in the RPL security research domain. Additionally, we illustrated the current challenges of reviewed studies and some security issues related to the RPL network.

As for this study’s limitations, first, this study is limited to a few selected databases, although they are considered the most reliable sample of sources related to the research topic of this study. Nevertheless, other indexed databases might also contain studies relevant to this topic. However, we omitted those resources due to subscription requirements, limiting our access to their databases.

Second, this SLR study is limited to ML, DL, and combined approaches that detect RPL-based attacks, as presented in Figure 3. In addition, this study provides a comprehensive review and critical analysis focusing only on the recent ML, DL, and combined ML- and DL-based approaches for detecting RPL-based attacks, along with existing benchmark datasets used to evaluate their effectiveness.

Third, there are many SLR studies related to this topic using different sets of research keywords (see Section 4.1). However, to the best of our knowledge, there are no standard keywords related to RPL and 6LoWPAN networks exist. Moreover, our search is limited to English-language studies with a restricted range of publication years (January 2016 to mid-2021).

Finally, we suggested some potential research directions that will serve as a solid foundation for future researchers in the RPL domain.

## Figures and Tables

**Table 1:** Comparison of our SLR With existing literature studies.

Ref. No. & Year	Type of Study	RPL Architecture	RPL Security and Threats	RPL-ML Technique	RPL-DL Technique	RPL Datasets
[16], 2017	Survey	✓	✗	✗	✗	✗
[36], 2019	Review	✓	✓	✗	✗	✗
[25], 2020	Survey	✗	✗	✗	✗	✗
[37], 2020	Systematic Review	✓	✗	✗	✗	✗
[15], 2020	Survey	✗	✗	✗	✗	✗
[38], 2021	Critical Review	✗	✗	✗	✗	✗
[39], 2021	SLR	✓	✓	✗	✗	✗
[40], 2021	SLR	✗	✗	✗	✗	✗
This study, 2022	SLR	✓	✓	✓	✓	✓

✓: Covered, ✗: Not covered, SLR: Systematic Literature Review.

**Table 2:** Summary of machine learning-based approaches.

Ref./Year	A1	A2	A3	A4	A5	A6	A7	Advantages and Limitations
[8], (2017)	NA	K-Mean Clustering, DT, Hybrid (K-Mean Clustering and DT)	NA	Real-time Simulation Dataset	WH	C++	DR	The proposed K-Mean Clustering and DT models obtain the best result in DR, while the hybrid (K-Mean Clustering and DT) approach gains the lowest FPR compared to the K-Mean Clustering and DT models. However, the proposed approach targeted only one attack, and the result of the other evaluation metrics was missing. Furthermore, no further information about the features linked to the detected attacks is available.
[44], (2019)	All features used	Self-Organizing Map (SOM)	6	Synthetic Dataset	HF, SH, VN	Contiki (Cooja simulator), Python, Wireshark	Number of Broadcast Messages, PRC, U-Matrix	The proposed SOM model classified the datasets’ samples effectively. However, the authors did not provide information about the collected dataset. Furthermore, the critical performance metrics, such as AC, Precision, TPR, and others, are not analyzed. Moreover, the proposed mechanism is not suitable for constrained devices.
[45], (2019)	NA	MLP-based ANN	NA	Real-time Simulation Dataset	DIS Flooding, VN	Contiki (Cooja simulator), Wireshark, Weka	AC, TPR, FPR, Precision, Recall, F-Measure, MCC, ROC Area, PRC Area	The proposed ANNIDS approach achieved the highest TPR, Precision, Recall, and F-Measure results and detected the DIS and VN attacks accurately. However, the authors did not mention the dataset and selected features used. Furthermore, no explanation of the produced outcomes and deployment strategy is available. Lastly, the proposed model is not suitable for constrained devices.
[46], (2019)	All features used	Ensemble classifiers (Boosted Trees, Bagged Trees, Subspace Discriminant, RUSBoosted Trees)	20	RPL-NIDDS2017 Dataset	SH, BH, Sybil, CID, SF, HF, Local Repair	MATLAB (2017), Python	AC, AUC	The proposed ensemble model (Boosted Tree) achieves the best result for AC in the case of hold-out and cross-validation. In addition, the ensemble (RUS Boosted) model gains the highest result of AUC. However, there is no evaluation result for the other metrics and no comparison with the other traditional classifiers. The authors also do not provide information about the deployment strategy.
[47], (2019)	Pairwise Correlation	RF, NB, J48 Classifier	21	Synthetic Dataset	HF, DIS Flooding, IV, DRA	Python, Contiki (Cooja Simulator)	AC, Recall, Precision	The proposed framework reported that the RF classifier achieves the highest processing time, AC, Recall, and Precision results. In addition, the author clarified that the dimensional reduction technique significantly reduced energy consumption and processing time. However, the other evaluation metrics, such as packet loss and energy consumption, are not covered. Furthermore, the authors used a small number of network nodes and no further information about the availability of the generated multi-class dataset.
[48], (2019)	All features used	GP	50	Synthetic Dataset	HF, VN	Contiki (Cooja simulator)	TPR, FPR, AC	The proposed central architecture obtained the best AC result during 500 ms and 5000 ms. Moreover, the authors reported that the distributed architecture achieves high AC results with the help of network nodes. However, the proposed approach suffered from a single point of failure and used the network nodes for monitoring, adding more computational overhead and consuming the power resources. Furthermore, the proposed work does not provide information about the collected dataset.
[49], (2019)	All features used	NB, DT, LR, ANNs, EM Clustering	20, 23, 49, 41	NIDDS2017, WSN-DS, UNSW NB15, KDD99	SH, BH, HF, CID, Local Repair, Sybil, SF	MATLAB (R2017a), Weka Software (version 3.9)	AC, FPR	The proposed DT model achieved the best result of AC and FPR. In addition, EM Clustering registered the lowest result of AC. However, the authors did not use feature selection techniques and did not consider other vital analysis parameters like PDR, PRC, E2E Delay, etc. Furthermore, the deployment strategy for the proposed work was missing.
[50], (2019)	NA	K-NN	NA	NA	Rank	Contiki (Cooja simulator)	E2E Delay, PDR, TPR, FPR	The proposed mechanism results obtained significant reports that PDR and TPR improved after deploying the proposed mechanism compared to regular networks under Rank attack. Meanwhile, the results of the E2E delay and FPR declined after the proposed mechanism was spread throughout the network. However, the authors considered only one type of attack and ignored others. Furthermore, the proposed mechanism uses one metric (i.e., rank) to calculate the distance between static nodes and does not consider the mobility of other nodes. Meanwhile, the nodes participate in the detection process, creating additional overhead in the network.
[51], (2019)	NA	Kernel Density Estimation	NA	Real-time Simulation Dataset	HF, VN, BH	Contiki O.S. (Cooja simulator), Python	TPP, FPR, UDP Flow	The proposed approach achieved a significant average TPR result of detecting all types of attacks with different topologies. However, the result of the critical parameters, such as accuracy, precision, PDR, and E2E Delay, is not analyzed. Furthermore, the authors did not give details about the availability of the collected dataset, and the extracted features are not sufficient to detect other types of attacks.
[24], (2020)	All features used	FFNN	14	Synthetic Dataset	WP	Contiki (Cooja simulator)	AC, Precision, Recall, F-Measure, PRC	The devised FNN model achieved a significant result in terms of Accuracy Precision, Recall, and F-Score. Furthermore, the presented work identified the zone that launches the attack. However, the authors used few nodes, and their approach is limited to detecting one type of attack. Furthermore, no details were available about the availability of the dataset and the type of deployment strategy.
[3], (2020)	NA	Neural Network	27	Synthetic Dataset	WP, HF, VN	Contiki (Cooja simulator)	AC, DR, FPR	The proposed approach reported high results for detecting invoking attacks for binary and multi-class classification. In addition, the produced link-layer features decreased the FPR and slightly increased the DR of the VN attack. However, the authors used few nodes during the simulation and no details about the availability of the dataset. Moreover, other critical parameters, such as PDR, E2E delay, and PRC, are not available. Additionally, the deployment strategy for this work has not been provided.
[52], (2020)	GP	Threshold Statements	53	Synthetic Dataset	HF, VN, SH, BH	Contiki (Cooja simulator)	AC, TPR, FPR, PDR	The presented framework revealed the best results from HF and VN attacks during the 2000 seconds. Meanwhile, the highest AC result for detecting SH and BH attacks was obtained at 1000 s. Furthermore, the results of PDR and TPR have improved. However, when the number of network nodes raised, the PDR declined, which led to inaccurate detection of the attack. Moreover, the authors evaluated their work with small numbers of network nodes. Additionally, no details were available on the deployment strategy of the presented work.
[10], (2020)	NA	DT, SVM, Bernoulli and LR	5	Synthetic Dataset	VN	Contiki (Cooja simulator)	AC, Precision, Recall, Specificity	The introduced framework reported significant AC, Precision, Recall, and Specificity outcomes. However, there is no evaluation of the other critical metrics, such as PDR, PRC, and E2E delay. Furthermore, this work is limited to only one attack.
[21], (2020)	NA	Artificial Intelligence-based Packet Drop Ratio	NA	Real-time Simulation Dataset	SF	Contiki (Cooja simulator)	PDR, E2E Delay, DR	The proposed technique obtained significant results in terms of TPR and FPR. After implementing the proposed approach in the network, the result of the E2E delay improved. However, the proposed mechanism failed to improve the PDR. Furthermore, the proposed approach addressed one type of attack with small numbers of networks nodes.
[53], (2020)	NA	One-Class SVM	NA	Real-time Simulation Dataset	Rank	Contiki (Cooja simulator)	PRC, Anomaly DR	The proposed ML approach reported good results in terms of anomaly detection rate. However, this work deals with one type of attack, and the presented work employs a small number of nodes. Furthermore, the outcomes of the other metrics, such as PDR, E2E delay, and PLR, are not computed.
[54], (2020)	NA	DT	11	Synthetic Dataset	HF, VN	Contiki (Cooja simulator), Python, Wireshark	PRC, Precision, Recall, AC, FPR	The presented work obtained promising results for AC and FAR. However, this work targeted only one type of attack, with no details about the other critical evaluation parameters, such as packet loss and E2E delay. Furthermore, the presented analysis factors cannot detect other types of attacks. Moreover, the proposed work suffers from high PRC.
[55], (2020)	GP	DT, SVM, Bayesian Classifiers	5	Synthetic Dataset	SH	Contiki (Cooja simulator), RapidMiner	DR, FPR	The proposed Bayesian model achieved the highest DR after applying the alarm verification method. Meanwhile, the DT classifier reported the highest level of Precision compared to the others. However, the proposed approach suffers from high FPR, and no further information about the deployment strategy is available. Moreover, the proposed work is limited to detecting only one type of attack.
[56], (2021)	NA	ANN	23,22, 32,28	Synthetic Dataset	HF, DRA, IV	Contiki (Cooja simulator), Python, Wireshark	PRC, Number of Packets, AC, Recall, Precision F- Score, MCC	The introduced AIEMIA approach reached the maximum result of AC using the hold-out validation technique. The authors reported good outcomes for the other performance measures such as PRC, Number of Packets, etc. However, no information about the collected features is available. Furthermore, the author used a small network size for the dataset collection.
[57], (2021)	Information Gain Algorithm	ANN	8	IRAD Dataset	VN, DRA, HF	NA	AC, Loss, Precision, Recall, F1-Score, Support	The proposed MLRPL model attained high AC and other evaluation metrics in binary and multi-class results. In addition, the performance of the proposed approach exceeds other existing approaches. However, the proposed model takes a long training time to achieve the best result. Furthermore, the authors did not provide details about the deployment strategy for the network and the software specifications.
[31], (2021)	Step Forward Feature Selection	Gradient Boosting ML	11	Synthetic Dataset	VN	Contiki (Cooja simulator), Python, Wireshark	AC, FPR, TNR, Precision, Recall, F1-Score, ROC	The devised ML-LGBM model achieved high AC, Precision, F-Score, TNR, and FNR. In addition, the presented work exceeded the approaches in terms of training time, testing time, model size, and other metrics. However, only one type of attack is detectable in this work, and no details are available on the generated dataset’s availability. Additionally, the authors used small network nodes during the dataset’s generation process.
[59], (2021)	Principle Component Analysis	SVM	NA	Real-time Simulation Dataset	VN, Rank, DoS	Contiki (Cooja simulator),Python	AC, Recall, Precision, F Measure, PDR, Control Overhead, Energy Consumption	The proposed MLRP model achieved significant results and improved the PDR of the base-RPL. The result also reported a high detection of targeted attacks with implementing the PCA technique. However, despite the significance of PDR, the proposed approach suffers from low PDR, which requires more improvement. Furthermore, there is no information about the dataset’s availability and the number of selected features regarding the generated dataset. Moreover, the authors used few nodes during the data generation stage.
[60], (2021)	NA	Ant Colony Optimisation	NA	NA	RPL Attacks	NA	Throughput, Number of Packets, Response Time	The proposed security mechanism improved the quality of service and routing process. Furthermore, the presented work proves its efficiency in enhancing the throughput and number of packets compared to the SecTrust-RPL approach. However, the authors did not specify the type of targeted attacks and the programs used. Furthermore, there are no details about the deployment strategy.
[7], (2021)	NA	SVM	NA	IDC and EDC Datasets	Heart, Temperature Level, Flooding, VN, Rank	Contiki (Cooja simulator), Python	Detection AC, PRC	The introduced anomaly detection approach obtained high detection of e-health related data and network attacks. In addition, the proposed approach provided low-cost management and accurate decisions by utilizing a standard management program with reliable features. However, the proposed approach identifies other attacks, such as Flooding and event attacks, with a low DR. Furthermore, the authors did not present details about the feature selection technique and availability of the used dataset.

A1: Feature Selection Technique, A2: Classification/ Detection Mechanism(s), A3: No. of Features, A4: Dataset, A5: Relevant Attack(s), A6: Software/Tools, A7: Evaluation Metrics, NA: Not Available, DT: Decision Tree, RF: Random Forest, NB: Naïve Bayes, GP: Genetic Programming, LR: Logistic Regression, ANN: Artificial Neural Network, SVM: Support Vector Machine, FFNN: Feedforward Neural Network, K-NN: K-Nearest Neighbor, MLP: Multi-Layer Perceptron, SOM: Self Organizing Map, WH: WormHole, SH: SinkHole, BH: BlackHole, CID: Clone ID, SF: Selective Forwarding, HF: Hello Flooding, IV: Increased Version, DRA: Decreased Rank, VN: Version Number, DoS: Denial of Service, WP: Worst Parent, DR: Detection Rate, AC: Accuracy, AUC: Area Under Curve, TPR: True Positive Rate, FPR: False Positive Rate, E2E: End to End, PDR: Packet Delivery Ratio, FNR: False Negative Rate, TNR: True Negative Rate, PRC: Power Consumption, EM: Expectation-Maximization, ROC: Receiver Operating Characteristic, MCC: Matthew’s Correlation Coefficient.

**Table 3:** Summary of deep learning-based approaches.

Ref./Year	A1	A2	A3	A4	A5	A6	A7	Advantages and Limitations
[58], (2018)	DT, Pearson Coefficient, Histogram	MLP-based ANN	17	IRAD Dataset	HF, VN, DRA	Contiki (Cooja simulator), Python, Wireshark	Precision, Recall, F1 Score, AUC, Loss, AC	The proposed DL model obtained the highest results in identifying HF attacks. The presented approach detects the other attacks significantly. In addition, this work provides a comprehensive analysis of the proposed dataset and different scenarios for each type of attack with diverse sizes of networks. However, the analysis of other critical parameters, such as PDR, E2E delay, and PRC, has not been provided. Furthermore, no information about the deployment strategy is available. Additionally, the proposed approach is not suitable for dynamic network traffic.
[63], (2017)	Perceptual Learning Model	DNN, DBN	8	NA	BH, Opportunistic Service, DDoS, SH, WH	Contiki (Cooja simulator), Python	Precision, TPR, F1-Score, P-R Curves	The presented mechanism achieved a significant attack detection result and a good Precision outcome. However, they did not cover other critical parameters, such as PDR, E2E delay, and PRC. Furthermore, the authors did not provide details about the used dataset and selected features.
[1], (2020)	One-R, Chi-Square, Weighted RF	CNN	15	IoT Routing Dataset	HF, SF, SH, WH, VN	Contiki (Cooja simulator), RapidMiner	Model Accuracy, Loss Function, AC, Error, Precision, Recall, F-measure, Correlation, Logistic Loss	The proposed model achieved a significant result for detecting attacks with low error and loss rates. Furthermore, the proposed CNN model reduced the PRC and maintained the stability of the IoT network. However, it required an extended processing time to reach its best result and failed to explain the deployment strategy. Furthermore, the authors did not disclose the dataset and selected features used. Moreover, there are no critical parameters, such as PDR, PRC, and E2E delay details.
[62], (2020)	All features used	MLP	NA	Synthetic Dataset	Rank	Contiki (Cooja simulator), Wireshark	TPR, FPR, macro AVG, Weighted Avg, AC, Precision, Recall, F-scores	The presented MLP algorithm achieved a high AC result for different scenarios of attacks. Furthermore, the result reported that this approach is capable of sorting and distinguishing various kinds of attacks. However, despite all these benefits, no details about the used features or availability of the generated dataset are available.
[65], (2021)	NA	LSTM, Graph CNN	7	Synthetic Dataset	DRA, IR, WP	Contiki (Cooja simulator), Ethereum Client (Geth)	Time of Arrival, AC, Precision, Recall, F1-Score, Model Probabilities	The designed DL framework achieved a high result of accuracy for different scenarios. Furthermore, the result reported that this approach is capable of sorting and distinguishing between various kinds of attacks. However, the proposed framework is not suitable for low-power devices and creates additional overhead on the network. Additionally, the authors did not provide details about the deployment strategy, and no information is available on other critical parameters, such as PDR, E2E delay, and PRC.
[5], (2021)	All features used	Auto Encoder and DNN	19	Synthetic Dataset	CID	Contiki (Cooja simulator), Wireshark	AC, F-Score, Total time	The introduced (SAE + DNN) framework provided high detection accuracy in detecting CID attacks. In addition, the authors compared their work with other existing approaches and exceeded them in terms of effectiveness. However, the proposed work is limited to detecting only one type of attack, and there was no information about the availability of the dataset. Moreover, the author used a small number of nodes during the data collection step. Finally, no analysis of the other critical parameters, such as PDR, E2E delay, and PRC, are available.

A1: Feature Selection Technique, A2: Classification/ Detection Mechanism(s), A3: No. of Features, A4: Dataset, A5: Relevant Attack(s), A6: Software/Tools, A7: Evaluation Metrics, NA: Not Available, HF: Hello Flooding, VN: Version Number, CID: Clone ID, DRA: Decreased Rank, SH: SinkHole, BH: BlackHole, DDoS: Distributed Denial of Service, WH: WormHole, WP: Worst Parent, IR: Increased Rank, SF: Selective Forwarding, DT: Decision Tree, MLP: Multi-Layer Perceptron, DL: Deep Learning, DBN: Deep Belief Network, SAE: Sparse Auto Encoding, DNN: Deep Neural Network, ANN: Artificial Neural Network, CNN: Convolutional Neural Network, One-R: One Rule, RF: Random Forest, LSTM: Long Short-Term Memory, AUC: Area Under Curve, AC: Accuracy, DR: Detection Rate, PRC: Power Consumption, TPR: True Positive Rate, FPR: False Positive Rate, E2E: End to End.

**Table 4:** Summary of combined ML- and DL-based approaches.

Ref./Year	A1	A2	A3	A4	A5	A6	A7	Advantages and Limitations
[30], (2020)	NA	RNN, SVM, LR	5	Synthetic Dataset	HF	Contiki (Cooja simulator), Python	PDR, ERC, Average Delay, AC, MSE, MAE, RMSE	The proposed hybrid model achieved significant PDR and average delay for attack identification with a different set of features. In addition, the best result for RMSE, MSE, MAE, and AC is obtained in the third scenario. Furthermore, the GRU attains the best result for AC compared to SVM and LR in most cases. However, the presented work targeted only one type of attack. Furthermore, the presented work was tested with a few nodes during data collection steps and suffered from scalability issues when the number of nodes was raised. Moreover, this work lacked information about the availability of the used dataset and the used feature selection technique.
[66], (2020)	NA	NB, SVM, MLP, RF, ZeroR	24	Synthetic Dataset	Rank, VN, Rank + Sybil, Rank + BH, Decreased Path Metric	Contiki (Cooja simulator), Weka	RMSE, MAPE, ROC Average, Correctly Classified Instances, Balancing Technique Average	The presented approach significantly detected the invoked attacks in both objective functions (OF0 and MRHOF). In addition, the overall performance of the presented approach reported that the voting (MLP and RF) achieved excellent results compared to other approaches. Furthermore, the SMOTE-MLP model achieved good results in some experiments. However, the devised work could not analyze other critical parameters, such as AC, Precision, Recall, PDR, E2E delay, and PRC. Furthermore, there was no information about the deployment strategy of the presented work and the availability of the generated dataset.
[67], (2020)	CS + Dagging + base learner BLR	SVM, DL, Fuzzy Unordered Rule Induction Algorithm	12, 15	IoT Routing Dataset	IoT Routing	Contiki (Cooja simulator), Weka	AC, Error, F-Measure	This study reported that the AC, Error, and f-Measure of (CS algorithm using Dagging with BLR) model is better than the CS algorithm with BLR model. In addition, the CNN model achieved better results in all metrics measured than other classification algorithms. However, the presented approach did not provide information about the availability of the dataset and the deployment strategy. Furthermore, the study did not analyze other critical metrics, such as PDR and E2E delay. Furthermore, the authors did not identify the type of targeted attack in this work.
[32], (2021)	RF	MLP, KNN, AdaBoost, RF, GNB, LR, DT	21	Synthetic Dataset	SH, DIO Suppression, BH, SF, Sybil, DIS Flooding	NetSim, Python	AC, Precision, Recall, AUC, ROC, F1-Score	The proposed DT achieved the best AC, Precision, and F-Score results. In addition, the LR, GNB, and MLP achieved the highest results in Recall value, and the RF model achieved the best result in AUC. However, the authors did not introduce analysis in terms of PDR, E2E delay, and PRC. Furthermore, there was no information about the availability of the dataset nor details about the deployment strategy. Moreover, the proposed ML algorithms create additional overhead, and then they are not suitable for constrained devices.
[35], (2021)	RF, PC	DT, RF, K-NN, NB, MLP, LR, Sequential DL model	7, 10, 7, 10, 6, 7, 13	Synthetic Dataset	DRA, BH, SH, HF, SF, VN	Contiki (Cooja simulator), Python, Wireshark	AC, Precision, Recall, F1-Score, Fitting Time	The proposed model achieved significant results in all the used metrics for detecting the attack in both two-class and multi-class classifications. In addition, the RF classifier achieved the lowest fitting time. Furthermore, the presented work introduced the RF-IDSR approach, which provides fault tolerance and intrusion tolerance in Industry 4.0 networks. However, no information about the availability of the dataset and the deployment strategy is available. Furthermore, there was no analysis of the other critical metrics, such as PDR, PRC, and E2E delay.
[68], (2021)	PSO	NB, SVM, RF, K-NN, False MLP	15	Synthetic Dataset	HF, WH, SH	Contiki (Cooja simulator), Python, Wireshark	AC, Precision, Recall, TPR, FPR	The RF algorithm achieved the best result in detecting the invoked attacks. However, the proposed approach did not provide information about the deployment strategy or details about the generated dataset’s availability. Moreover, the proposed technique added more computational overhead to the network, consuming power resources. Furthermore, there was no analysis of the other crucial parameters, such as PDR, E2E delay, and PRC.

A1: Feature Selection Technique, A2: Classification/ Detection Mechanism(s), A3: No. of Features, A4: Dataset, A5: Relevant Attack(s), A6: Software/Tools, A7: Evaluation Metrics, NA: Not Available, CS: Cuckoo Search, DT: Decision Tree, BLR: Bayesian Logistic Regression, RF: Random Forest, CNN: Convolutional Neural Network, RNN: Recurrent Neural Network, LR: Logistic Regression, SVM: Support Vector Machine, DL: Deep Learning, MLP: Multi-Layer Perceptron, NB: Naïve Bayes, K-NN: K-Nearest Neighbor, PSO: Particle Swarm Optimization, PC: Pearson Correlation, SMOTE: Synthetic Minority Oversampling Technique, PDR: Packet Delivery Ratio, AC: Accuracy, MSE: Mean Square Error, MAE: Mean Absolute Error, RMSE: Root Mean Square Error, TPR: True Positive Rate, FPR: False Positive Rate, GNB: Gaussian Naïve Bayes, AUC: Area Under Curve, ROC: Receiver Operating Characteristic, MAPE: Mean Absolute Percentage Error, E2E: End to End, ERC: Energy Consumption, PRC: Power Consumption, HF: Hello Flooding, VN: Version Number, BH: BlackHole, SH: SinkHole, SF: Selective Forwarding, DRA: Decreased Rank, WH: WormHole.

## Data Availability

Not applicable.
